# Clinical features of obscure gastrointestinal bleeding undergoing capsule endoscopy: A retrospective cohort study

**DOI:** 10.1371/journal.pone.0265903

**Published:** 2022-03-24

**Authors:** Yuga Komaki, Shuji Kanmura, Kazuki Yutsudo, Kosuke Kuwazuru, Fukiko Komaki, Akihito Tanaka, Hidehito Maeda, Shiho Arima, Shiroh Tanoue, Fumisato Sasaki, Shinichi Hashimoto, Masahisa Horiuchi, Akio Ido

**Affiliations:** 1 Digestive and Lifestyle Diseases, Kagoshima University Graduate School of Medical and Dental Sciences, Kagoshima, Japan; 2 Hygiene and Health Promotion Medicine, Kagoshima University Graduate School of Medical and Dental Sciences, Kagoshima, Japan; 3 Department of Gastroenterology, Izumi General Medical Center, Kagoshima, Japan; 4 Department of Gastroenterology, Kagoshima City Hospital, Kagoshima, Japan; School of Digestive & Liver Diseases, Institute of Post Graduate Medical Education & Research, INDIA

## Abstract

**Background:**

Capsule endoscopy has been widely used to investigate obscure gastrointestinal bleeding (OGIB) in the small intestine since its approval in 2001. However, the clinical features of OGIB remain unclear.

**Aim:**

We retrospectively examined the clinical features and risk factors of OGIB in patients who underwent capsule endoscopy in our hospital.

**Methods:**

We included 420 of the 431 patients who underwent capsule endoscopy from June 2014 to May 2021, in whom the small intestine could be observed. We retrospectively compared the clinical features and treatment of OGIB cases, with or without active small bowel bleeding (n = 173), with other cases (n = 247). Patient sex, age, diabetes mellitus, and heart failure histories were matched for the analysis.

**Results:**

The male/female ratio was 247/173 and the average age was 51.54 years. In multivariate analysis, the use of direct oral anticoagulants was significant (*P* = 0.016), and vascular lesions (*P* = 0.018) were observed in OGIB cases. When OGIB cases with and without active small bowel bleeding were compared, serum albumin level was lower in cases with active bleeding (*P* = 0.031). When treatment of OGIB cases were compared, those without vascular lesions could be treated conservatively (*P* = 0.0047). In the 1:1 propensity score matching analysis, serum creatinine level was elevated in cases of active bleeding (*P* = 0.029), and cases without vascular lesions were treated conservatively (*P* = 0.010).

**Conclusions:**

Use of direct oral anticoagulants is frequently associated with OGIB. OGIB patients without vascular lesions may be treated conservatively.

## Introduction

Gastrointestinal (GI) bleeding is a common condition in clinical practice with significant morbidity and mortality. In 80–90% of cases, the bleeding lesion is found on upper or lower endoscopy, although nearly half of the patients with negative endoscopy findings (5–10%) have continued or recurrent bleeding [1]. Obscure gastrointestinal bleeding (OGIB) is defined as GI bleeding, either overt or occult, which remains undiagnosed with respect to the underlying etiology, despite upper and lower gastrointestinal endoscopies [2]. Occult OGIB is defined as obscure bleeding without macroscopic blood in fecal excrement. When upper endoscopy and colonoscopy fail to find a bleeding focus, further investigations are recommended [3]. Capsule endoscopy is the most valuable modality for OGIB diagnosis [4–6]. In a previous report, it has been clarified that the high diagnostic yield of capsule endoscopy supports its role as the first-line investigation in patients with OGIB [7]. Moreover, there have been several reports investigating the risk factors for small bowel bleeding and their correlation with comorbidities or specific drugs, such as non-steroidal anti-inflammatory drugs (NSAIDs), thienopyridines, anticoagulants, and proton pump inhibitor (PPIs) [8, 9]. However, regarding small intestinal bleeding, there are few papers that have comprehensively examined background factors, including not only comorbidities, drugs, and lifestyle-related diseases, but also characteristic findings of the small intestine. In short, the clinical features of OGIB remain unclear. Therefore, we decided to retrospectively examine the clinical features and risk factors of OGIB in patients who underwent capsule endoscopy in our hospital and identify significant associations.

## Materials and methods

**Fig 1:**
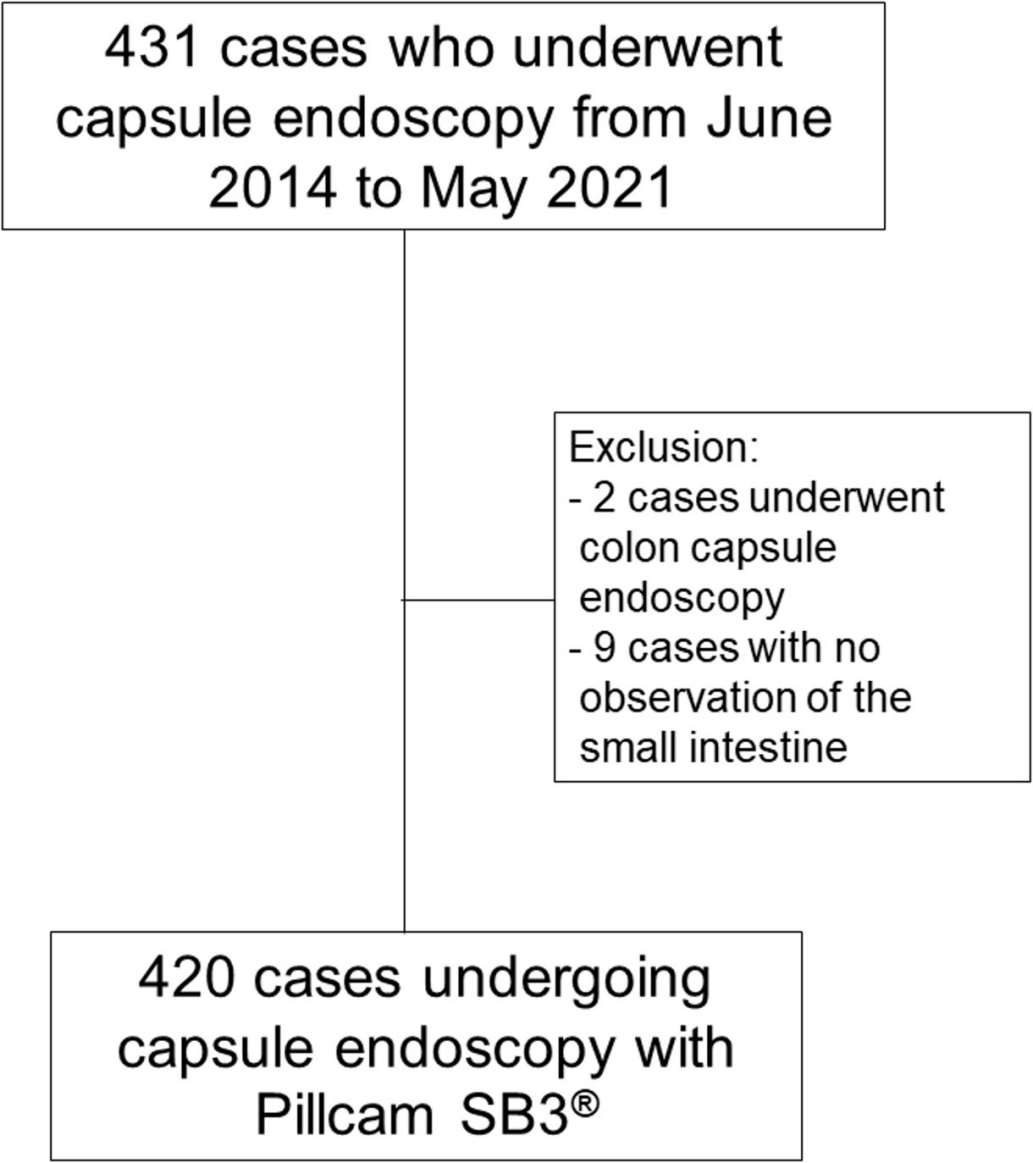
Flow chart of data analysis. Of 431 patients who underwent capsule endoscopy, nine cases in which the small intestine could not be confirmed at all, and two cases in which colon capsule endoscopy was performed, were excluded. Finally, 420 patients who underwent capsule endoscopy with Pillcam SB3^®^ were selected.

This was an observational, retrospective study of patients who underwent capsule endoscopy between 1 June 2014 and 31 May 2021. During the study period a total of 431 cases underwent capsule endoscopy with Pillcam SB3^®^ and 420 cases were included in the analysis. Excluded cases were those who had undergone colon capsule endoscopy or those in whom the small intestine could not be observed (Fig 1). Study patients included both adults and children who underwent capsule endoscopy.

The study used an “opt-out” option to obtain patient consent after being approved by the Ethics Committee at Kagoshima University Hospital (institutional review board 190060) on 19 August 2019 and was undertaken on a priori defined protocol [10].

The primary endpoint was the clinical features of patients with and without OGIB, who underwent capsule endoscopy. The clinical features included sex; age; smoking; current medications such as antithrombotic drugs, NSAIDs, probiotics and antacids; and comorbidities such as hypertension, diabetes mellitus, dyslipidemia, cerebrovascular disease, and heart disease. The secondary endpoint was the difference in clinical features between the groups with and without active bleeding, and according to the location of the lesion in the small bowel. In addition, we compared the clinical features of cases of OGIB that received different treatments. Inpatient and outpatient charts were reviewed. OGIB was defined as GI bleeding that persists or recurs without an obvious etiology after standard endoscopic examination (routine upper endoscopy and colonoscopy) [11]. Our analyses included cases based on this general definition, as well as cases with no obvious bloody stool but with a positive fecal occult blood test. For each analysis, additional analyses were performed separately for adults and children (defined as under 18 years old). Since the number of cases was as small as 11, OGIB in children could not be examined.

For adjustment of baseline characteristics, we performed propensity score matching among cases of OGIB. We adjusted for sex, age, diabetes mellitus, and heart failure as patient characteristics.

### Statistical analysis

Continuous variables were expressed as mean ± standard deviation (SD) and were examined by dividing them into large and small median values. Comparisons between categorical variables were analyzed using Fisher’s exact test, because almost all the continuous variable factors showed non-normal distribution using Shapiro–Wilk test. A *P* value of <0.05 was considered statistically significant. The primary outcome was analyzed using univariate and multivariate comparisons among all patients that underwent capsule endoscopy. Univariate predictor variables with a *P* value <0.05 were included in the multivariate analysis. Each multivariate analysis was performed by a logistic regression analysis.

To reduce the effect of treatment-selection bias and potential confounding in this observational study, we adjusted for significant differences in the baseline characteristics of patients with propensity score matching. Patients were matched for age, sex, diabetes mellitus, and heart failure. For example, serving as a control, one patient who underwent capsule endoscopy for OGIB with active bleeding was matched to a patient who underwent capsule endoscopy for OGIB without active bleeding [12].

For all statistical analyses, data were analyzed by EZR version 1.54 (Saitama Medical Center, Jichi Medical University, Saitama, Japan) [13], which is a graphical user interface for R (The R Foundation for Statistical Computing, version 2.13.0, Vienna, Austria).

## Results

### Patient characteristics

The characteristics of patients included in our analysis are shown in Table 1. Among the 420 cases who underwent capsule endoscopy (247 men and 173 women), 147 cases were due to OGIB, 133 were due to inflammatory bowel disease, 70 were due to tumors of the small intestine, and 70 were due to diarrhea or stomach-ache, which were the main reasons for investigation. The mean age was 51.54. The entire small intestine was observed in 362 cases. There were 51 pediatric patients under 18 years old including 5 patients under 10 years old who underwent capsule endoscopy in our study; among these 5 patients, 4 patients, aged 2 to 5 years, had a capsule endoscopy inserted into the duodenum using an endoscope during upper gastrointestinal endoscopy under general anesthesia. The fifth patient, who was 8 years old, was able to take the capsule endoscope himself as an adult and perform the examination.

**Table 1:** Patient background information.

	n = 420 ^†^
Age (years), mean (range)	51.54 (2–89)
Sex, male/female	247/173
Observation of the entire small intestine, yes/no (percentage of “yes”)	362/58 (86.2%)
Main indications for CE, n (%)	
Suspected OGIB	147 (35.0%)
Follow-up of patients with IBD (e.g., Crohn’s disease and Behçet’s disease)	133 (31.7%)
Suspected small intestine tumor	70 (16.7%)
Diarrhea, stomach-ache, and others	70 (16.7%)
Presence of erosion or ulcer in the small intestine, n (%)	209 (49.8%)
Presence of vascular lesions in the small intestine (Types 1a/1b/2a/2b/3/4) ^‡^, n	36 (15/15/2/1/0/3)
Current or former smoker, n (%)	143 (34.0%)
Current warfarin user, n (%)	24 (5.7%)
Current DOAC user, n (%)	19 (4.5%)
Current Dabigatran user, n (%)	0 (0%)
Current Rivaroxaban user, n (%)	1 (0.2%)
Current Apixaban user, n (%)	7 (1.7%)
Current Edoxaban user, n (%)	11 (2.6%)
Current Aspirin user, n (%)	34 (8.1%)
Current Thienopyridines user, n (%)	15 (3.6%)
Current NSAIDs user, n (%)	27 (6.4%)
Current probiotics user, n (%)	58 (13.8%)
Current PPI or P-cab user, n (%)	148 (35.2%)
Hypertension, n (%)	134 (31.9%)
Diabetes mellitus, n (%)	52 (12.4%)
Dyslipidemia, n (%)	68 (16.2%)
Cerebral hemorrhage or infarction, n (%)	39 (9.3%)
Ischemic heart disease, n (%)	33 (7.9%)

CE, capsule endoscopy; OGIB, obscure gastrointestinal bleeding; IBD, inflammatory bowel disease; DOAC, direct oral anticoagulant; NSAIDs, non-steroidal anti-inflammatory drugs; PPI, proton pomp inhibitor; P-CAB, potassium-competitive acid blocker.

† Total number of capsule endoscopies

‡ Yano-Yamamoto classification.

### Comparison of clinical features in patients who underwent capsule endoscopy

As shown in Table 2, among 420 patients who underwent capsule endoscopy, univariate analysis demonstrated that age at capsule endoscopy ≥ 59.15 years (*P* <0.0001), presence of vascular lesions (*P* <0.0001), current warfarin usage (*P* = 0.00015), current direct oral anticoagulant (DOAC) usage (*P* <0.0001), current aspirin usage (*P* = 0.0057), current PPI or potassium-competitive acid blocker (P-CAB) usage (*P* <0.0001), platelet count at capsule endoscopy ≥ 249 /μL x10E3 (*P* = 0.0032), prothrombin time-international normalized ratio (PT-INR) at capsule endoscopy ≥ 1.040 (*P* = 0.013), blood urea nitrogen (BUN) at capsule endoscopy ≥ 12.70 mg/dl (*P* <0.0001), total protein (TP) at capsule endoscopy ≥ 6.70 g/dl (*P* <0.0001), serum albumin at capsule endoscopy ≥ 3.80 g/dl (*P* <0.0001), each comorbidity of hypertension, dyslipidemia, heart failure and atrial fibrillation (*P* <0.0001, = 0.0044, <0.0001, <0.0001, respectively), and each comorbidity or anamnesis of cerebral hemorrhage, cerebral infarction, ischemic heart disease and valvulitis (*P* = 0.0096, 0.0013, 0.00017, 0.00024, respectively) were associated with a significantly increased risk of OGIB. Multivariate analysis revealed that the presence of vascular lesions (Odds ratio (OR) 15.50, 95% confidence interval (CI) 1.61–150.00, *P* = 0.018) and current DOAC usage (OR 16.70, 95% CI 1.68–165.00, *P* = 0.016) remained risk factors for OGIB.

**Table 2:** Comparison of clinical features in patients who underwent capsule endoscopy, identified by univariate and multivariate analysis.

Factors	Reason for CE		Univariate			Multivariate		
	OGIB (n = 173)	Ex. OGIB ^‡^ (n = 247)	OR	95% CI	*P* *	OR	95% CI	*P* *
Age ≥ 59.15 years, yes/no (mean±SD) ^†^	120/53 (61.00±20.43)	89/158 (45.55±22.11)	4.00	2.60–6.22	<0.0001	1.89	0.77–4.65	0.17
Sex, male/female	102/71	145/102	0.99	0.65–1.50	1.00			
Presence of erosion or ulcer, yes/no	84/89	125/121 ^§^	0.91	0.61–1.37	0.69			
Presence of vascular lesions, yes/no	34/139	2/244 ^§^	29.65	7.41–256.78	<0.0001	15.50	1.61–150.00	0.018
Current or former smoker, yes/no	67/92 ^§^	76/147 ^§^	1.41	0.91–2.19	0.13			
Current warfarin user, yes/no	19/154	5/241 ^§^	5.92	2.083–20.72	0.00015	3.68	0.60–24.70	0.15
Current DOAC user, yes/no	18/155	1/245 ^§^	28.29	4.38–1,183.34	<0.0001	16.70	1.68–165.00	0.016
Current Aspirin user, yes/no	22/151	12/234 ^§^	2.83	1.30–6.48	0.0057	1.98	0.48–8.070	0.34
Current Thienopyridines user, yes/no	8/165	7/239 ^§^	1.65	0.51–5.47	0.425			
Current NSAIDs user, yes/no	11/162	16/228 ^§^	0.97	0.39–2.29	1.00			
Current probiotics user, yes/no	19/152 ^§^	39/207 ^§^	0.66	0.35–1.23	0.20			
Current PPI or P-CAB user, yes/no	83/90	65/180 ^§^	2.55	1.66–3.94	<0.0001	0.41	0.16–1.040	0.062
WBC ≥ 5,590.00/μL, yes/no (mean±SD) ^†^	76/95 (5,730.47±2,830.64) ^§^	123/104 (6,396.62±2,997.68) ^§^	0.68	0.44–1.028	0.068			
Platelets ≥ 249.00/μL x10E3, yes/no (mean±SD) ^†^	70/100 (225.77±121.059) ^§^	128/99 (277.39±113.15) ^§^	0.54	0.35–0.83	0.0032	1.49	0.65–3.46	0.35
PT-INR ≥ 1.040, yes/no (mean±SD) ^†^	93/65 (1.21±4.43) ^§^	84/102 (1.047±0.12) ^§^	1.73	1.11–2.73	0.013	1.10	0.49–2.48	0.82
BUN ≥ 12.70 mg/dL, yes/no (mean±SD) ^†^	110/59 (19.97±16.69) ^§^	91/134 (12.43±5.75) ^§^	2.74	1.78–4.24	<0.0001	1.29	0.53–3.15	0.58
Cr ≥ 0.76 mg/dL, yes/no (mean±SD) ^†^	94/76 (1.34±1.81) ^§^	109/119 (0.83±0.60) ^§^	1.35	0.89–2.052	0.16			
BUN/Cr ≥ 15.80, yes/no (mean±SD) ^†^	93/77 (19.51±10.82) ^§^	102/122 (16.33±6.43) ^§^	1.40	0.92–2.13	0.11			
TP ≥ 6.70 g/dL, yes/no (mean±SD) ^†^	48/112 (6.12±0.99) ^§^	149/68 (6.84±0.98) ^§^	0.20	0.12–0.31	<0.0001	0.52	0.21–1.33	0.17
Alb ≥ 3.80 g/dL, yes/no (mean±SD) ^†^	49/112 (3.26±0.78) ^§^	145/71 (3.88±0.84) ^§^	0.22	0.13–0.34	<0.0001	0.59	0.23–1.51	0.27
Hypertension, yes/no	78/95	56/190 ^§^	2.78	1.79–4.35	<0.0001	1.070	0.42–2.75	0.89
Diabetes mellitus, yes/no	23/149 ^§^	29/216 ^§^	1.15	0.61–2.15	0.65			
Dyslipidemia, yes/no	39/133 ^§^	29/217 ^§^	2.19	1.25–3.86	0.0044	1.10	0.36–3.35	0.86
Cerebral hemorrhage (current or past), yes/no	7/165 ^§^	1/245 ^§^	10.34	1.31–469.24	0.0096	1.53E6	0.00-inf.	0.99
Cerebral infarction (current or past), yes/no	22/150 ^§^	10/236 ^§^	3.45	1.52–8.40	0.0013	1.25	0.28–5.67	0.77
Ischemic heart disease, yes/no	24/148 ^§^	9/237 ^§^	4.26	1.85–10.70	0.00017	1.79	0.41–7.96	0.44
Valvulitis (pre- and post-operative), yes/no	27/89 ^§^	6/100 ^§^	5.023	1.92–15.58	0.00024	1.90	0.39–9.13	0.42
Aortic stenosis (pre- and post-operative), yes/no	11/106 ^§^	4/103 ^§^	2.66	0.76–11.83	0.11			
Aortic stenosis (pre-operative), yes/no	7/110 ^§^	3/103 ^§^	2.18	0.48–13.40	0.34			
Heart failure, yes/no	29/143 ^§^	4/241 ^§^	12.15	4.15–48.58	<0.0001	3.52	0.47–26.60	0.22
Atrial fibrillation, yes/no	18/154 ^§^	4/241 ^§^	7.011	2.25–29.037	<0.0001	0.30	0.032–2.75	0.29

CE, capsule endoscopy; OGIB, obscure gastrointestinal bleeding; OR, odds ratio; CI, confidence interval; SD, standard deviation; DOAC, direct oral anticoagulant; NSAIDs, non-steroidal anti-inflammatory drugs; PPI, proton pomp inhibitor; P-CAB, potassium-competitive acid blocker; WBC, white blood cells; PT-INR, prothrombin time-international normalized ratio; BUN, blood urea nitrogen; Cr, creatinine; TP, total protein; Alb, albumin.

* Fisher‘s exact test

† Divided by median number

‡ Cases with inflammatory bowel disease, suspected small intestine tumor, diarrhea, stomach-ache and others without fecal occult blood or obvious bloody stool were included

§ Data excluding missing value.

To ascertain whether vascular lesions were associated with any clinical features, particularly DOAC use, univariate and multivariate analysis comparing clinical features was undertaken (S1 Table). Hemoglobin (Hb) was significantly lower (OR 0.15, 95% CI 0.028–0.84, *P* = 0.030) and heart failure coexisted (OR 5.00, 95% CI 1.080–23.10, *P* = 0.039) in cases with vascular lesions, after multivariate analysis. The univariate analysis showed that DOAC use was not associated with the presence of vascular lesions in the small intestine (OR 2.075, 95% CI 0.37–7.80, *P* = 0.22).

When only the adult cohort was examined, the multivariate analysis after the univariate analysis revealed that the presence of vascular lesions (OR 11.00, 95% CI 1.040–115.00, *P* = 0.046), current DOAC usage (OR 19.90, 95% CI 2.040–194.00, *P* = 0.010), low white blood cells (OR 0.37, 95% CI 0.15–0.94, *P* = 0.036) and low serum albumin (OR 0.29, 95% CI 0.090–0.90, *P* = 0.032) remained clinical features for OGIB (S2 Table). It was presumed that the white blood cells were low in the OGIB group because the groups other than OGIB contained many cases of enteritis and tumors. When only the pediatric cohort was examined, as shown in S3 Table, the multivariate analysis could not be performed because there was only one item that had a significant difference in the univariate analysis; the BUN / Cr value was significantly higher in the OGIB group in the univariate analysis (OR 6.24, 95% CI 1.085–66.97, *P* = 0.037).

### Comparison of clinical features of OGIB cases with and without active bleeding, and according to lesion location

Among 173 OGIB patients, univariate analysis demonstrated that the presence of vascular lesions (*P* = 0.036), low Hb <9.050 g/dL (*P* = 0.038), low platelet count <216.50/μL x10E3 (*P* = 0.038), elevated BUN ≥ 14.80 mg/dl and serum creatinine ≥ 0.80 mg/dl (*P* = 0.039, 0.013, respectively), low serum albumin <3.30 g/dL (*P* = 0.0079), and diabetes mellitus (*P* = 0.029) were associated with a significantly increased risk of active bleeding. Multivariate analysis showed that low serum albumin <3.30 g/dL at capsule endoscopy (OR 0.38, 95% CI 0.15–0.92, *P* = 0.031) was a significant risk factor for active bleeding. Elevated creatinine (OR 2.32, 95% CI 0.90–5.96, *P* = 0.081) was a risk factor for active bleeding (Table 3).

**Table 3:** Comparison of clinical features in cases of obscure gastrointestinal bleeding with and without active bleeding, identified by univariate and multivariate analysis.

Factors	Cases of OGIB		Univariate			Multivariate		
	With active bleeding (n = 37)	Without active bleeding (n = 136)	OR	95% CI	*P* *	OR	95% CI	*P* *
Age ≥ 66.38 years, yes/no (mean±SD) ^†^	24/13 (66.83±16.75)	63/73 (59.72±21.24)	2.13	0.95–4.96	0.063			
Sex, male/female	21/16	81/55	1.12	0.50–2.49	0.85			
Presence of erosion or ulcer, yes/no	18/19	66/70	1.0048	0.45–2.22	1.00			
Presence of vascular lesions, yes/no	12/25	22/114	2.47	0.98–6.065	0.036	2.020	0.79–5.17	0.14
Current or former smoker, yes/no	17/17 ^‡^	50/75 ^‡^	1.50	0.65–3.45	0.33			
Current warfarin user, yes/no	5/32	14/122	1.36	0.36–4.38	0.56			
Current DOAC user, yes/no	4/33	14/122	1.056	0.24–3.67	1.00			
Current Aspirin user, yes/no	7/30	15/121	1.87	0.59–5.43	0.26			
Current Thienopyridines user, yes/no	2/35	6/130	1.24	0.12–7.31	0.68			
Current NSAIDs user, yes/no	1/36	10/126	0.35	0.0079–2.62	0.46			
Current probiotics user, yes/no	3/34	16/118 ^‡^	0.65	0.12–2.48	0.77			
Current PPI or P-CAB user, yes/no	20/17	63/73	1.36	0.62–3.031	0.46			
WBC ≥ 5,080.00/μL, yes/no (mean±SD) ^†^	17/19 (5,372.22±2,654.71) ^‡^	69/64 (5,826.00±2,877.62) ^‡^	0.84	0.38–1.88	0.71			
Hb ≥ 9.050 g/dL, yes/no (mean±SD) ^†^	12/24 (8.58±2.49) ^‡^	73/62 (9.52±2.39) ^‡^	0.43	0.18–0.97	0.038	0.88	0.36–2.18	0.78
Platelets ≥ 216.50/μL x10E3, yes/no (mean±SD) ^†^	12/23 (179.75±108.31) ^‡^	73/61 (238.044±121.69) ^‡^	0.44	0.18–1.0056	0.038	0.68	0.28–1.62	0.38
PT-INR ≥ 1.075, yes/no (mean±SD) ^†^	20/15 (1.29±0.57) ^‡^	60/64 (1.18±0.39) ^‡^	1.42	0.63–3.28	0.45			
BUN ≥ 14.80 mg/dL, yes/no (mean±SD) ^†^	24/12 (28.61±21.12) ^‡^	62/72 (17.65±14.52) ^‡^	2.31	1.013–5.52	0.039	1.22	0.47–3.15	0.68
Cr ≥ 0.80 mg/dL, yes/no (mean±SD) ^†^	25/10 (2.048±2.66) ^‡^	62/71 (1.15±1.45) ^‡^	2.85	1.21–7.19	0.013	2.32	0.90–5.96	0.081
BUN/Cr ≥ 16.83, yes/no (mean±SD) ^†^	12/15 (21.22±12.74) ^‡^	63/71 (19.052±10.25) ^‡^	1.57	0.70–3.59	0.26			
TP ≥ 6.20 g/dL, yes/no (mean±SD) ^†^	13/22 (5.87±0.90) ^‡^	67/58 (6.19±1.013) ^‡^	0.51	0.22–1.18	0.13			
Alb ≥ 3.30 g/dL, yes/no (mean±SD) ^†^	11/24 (3.074±0.69) ^‡^	72/54 (3.31±0.79) ^‡^	0.35	0.14–0.81	0.0079	0.38	0.15–0.92	0.031
Hypertension, yes/no	21/16	57/79	1.81	0.82–4.075	0.14			
Diabetes mellitus, yes/no	9/27 ^‡^	14/122	2.88	0.99–8.052	0.029	1.18	0.38–3.65	0.78
Dyslipidemia, yes/no	12/24 ^‡^	27/109	2.0094	0.81–4.83	0.12			
Cerebral hemorrhage (current or past), yes/no	0/36 ^‡^	7/129	0.00	0.00–2.62	0.35			
Cerebral infarction (current or past), yes/no	5/31 ^‡^	17/119	1.13	0.30–3.52	0.78			
Ischemic heart disease, yes/no	8/28 ^‡^	16/120	2.13	0.72–5.94	0.11			
Valvulitis (pre- and post-operative), yes/no	9/17 ^‡^	18/72 ^‡^	2.10	0.71–6.031	0.19			
Aortic stenosis (pre-^‡^ and post-operative), yes/no	4/23 ^‡^	7/83 ^‡^	2.047	0.40–8.92	0.28			
Aortic stenosis (pre-operative), yes/no	3/24 ^‡^	4/86 ^‡^	2.66	0.36–16.92	0.35			
Heart failure, yes/no	10/26 ^‡^	19/117	2.35	0.87–6.097	0.077			
Atrial fibrillation, yes/no	5/32	13/122 ^‡^	1.46	0.38–4.79	0.55			

OGIB, obscure gastrointestinal bleeding; OR; odds ratio; CI, confidence interval; SD, standard deviation; DOAC, direct oral anticoagulant; NSAIDs, non-steroidal anti-inflammatory drugs; PPI, proton pomp inhibitor; P-CAB, potassium-competitive acid blocker; WBC, white blood cells; Hb, hemoglobin; PT-INR, prothrombin time-international normalized ratio; BUN, blood urea nitrogen; Cr, creatinine; TP, total protein; Alb, albumin.

* Fisher‘s exact test

† Divided by median number

‡ Data excluding missing values.

Similar to the entire cohort, when only the adult cohort was examined, the multivariate analysis after the univariate analysis revealed that low serum albumin <3.30 g/dL at capsule endoscopy (OR 0.40, 95% CI 0.17–0.91, *P* = 0.029) was a significant risk factor for active bleeding (S4 Table). Since the number of cases was as small as 11, OGIB in children could not be examined.

For lesion location, the risk of OGIB was not significantly different between the upper small intestine (duodenum and jejunum) and the ileum (S5 Table). However, when only the adult cohort was examined, the multivariate analysis after the univariate analysis revealed that current aspirin usage (OR 0.23, 95% CI 0.036–1.11, *P* = 0.047) did not pose a risk of bleeding in the jejunum compared to the ileum (S6 Table).

### Comparison of clinical features of OGIB cases receiving different treatments

As shown in Table 4, among 161 patients with OGIB due to small intestinal bleeding, univariate analysis demonstrated that the presence of vascular lesions (*P* <0.0001), low Hb (<9.050 g/dL), low platelet count (<216.50/μL x10E3) and elevated BUN (≥ 14.80 mg/dl) at capsule endoscopy (*P* = 0.0020, 0.014, 0.0088 respectively), and diabetes mellitus (*P* = 0.0016) were associated with invasive treatments such as endoscopic treatment, interventional radiology (IVR) and surgery, compared with conservative treatment. Multivariate analysis showed that absence of vascular lesions (OR 3.97, 95% CI 1.53–10.30, *P* = 0.0047) were significantly associated with conservative treatment.

**Table 4:** Comparison of clinical features of cases of obscure gastrointestinal bleeding receiving different treatments, identified by univariate and multivariate analysis.

Factors	Cases of OGIB ^‡^		Univariate			Multivariate		
	Conservative treatment (n = 131)	Endoscopic treatment, IVR, surgery (n = 30)	OR	95% CI	*P* *	OR	95% CI	*P* *
Age ≥ 66.38 years, yes/no (mean±SD) ^†^	62/69 (63.24±21.57)	20/10 (60.79±20.25)	2.21	0.91–5.73	0.069			
Sex, male/female	72/59	22/8	0.45	0.16–1.13	0.099			
Presence of erosion or ulcer, yes/no	68/63 ^§^	11/19	0.54	0.21–1.30	0.16			
Presence of vascular lesions, yes/no	16/115	14/16	6.19	2.34–16.66	<0.0001	3.97	1.53–10.30	0.0047
Current or former smoker, yes/no	52/72 ^§^	11/15 ^§^	1.015	0.39–2.59	1.00			
Current warfarin user, yes/no	13/118	4/26	1.39	0.31–5.0076	0.53			
Current DOAC user, yes/no	14/117	1/29	0.29	0.0066–2.058	0.31			
Current Aspirin user, yes/no	19/112	3/27	0.66	0.12–2.48	0.77			
Current Thienopyridines user, yes/no	8/123	0/30	0.00	0.00–2.56	0.35			
Current NSAIDs user, yes/no	11/120	0/30	0.00	0.00–1.71	0.22			
Current probiotics user, yes/no	16/113 ^§^	1/29	0.24	0.0056–1.71	0.20			
Current PPI or P-CAB user, yes/no	59/72	15/15	1.22	0.51–2.92	0.69			
WBC ≥ 5,080.00/μL, yes/no (mean±SD) ^†^	68/61 (5,925.85±3,048.73) ^§^	12/18 (5,048.67±1,966.39)	0.60	0.24–1.44	0.23			
Hb ≥ 9.050 g/dL, yes/no (mean±SD) ^†^	73/57 (9.54±2.43) ^§^	7/23 (8.33±2.44)	0.24	0.081–0.63	0.0020	0.92	0.37–2.28	0.85
Platelets ≥ 216.50/μL x10E3, yes/no (mean±SD) ^†^	73/56 (241.16±126.12) ^§^	9/20 (177.40±93.89) ^§^	0.35	0.13–0.87	0.014	0.38	0.14–1.030	0.058
PT-INR ≥ 1.075, yes/no (mean±SD) ^†^	59/59 (1.20±0.40) ^§^	16/14 (1.23±0.57)	1.14	0.47–2.78	0.84			
BUN ≥ 14.80 mg/dL, yes/no (mean±SD) ^†^	60/69 (18.50±15.43) ^§^	22/8 (27.93±21.57)	3.14	1.23–8.78	0.0088	1.59	0.57–4.42	0.37
Cr ≥ 0.80 mg/dL, yes/no (mean±SD) ^†^	62/66 (1.14±1.33) ^§^	19/10 (2.32±3.13) ^§^	2.014	0.82–5.25	0.11			
BUN/Cr ≥ 16.83, yes/no (mean±SD) ^†^	63/66 (19.050±9.075) ^§^	16/14 (21.18±15.70)	1.20	0.50–2.89	0.69			
TP ≥ 6.20 g/dL, yes/no (mean±SD) ^†^	63/59 (6.19±0.99) ^§^	12/15 (5.82±1.030) ^§^	0.75	0.29–1.88	0.53			
Alb ≥ 3.30 g/dL, yes/no (mean±SD) ^†^	61/59 (3.26±0.77) ^§^	15/15 (3.22±0.79)	0.97	0.40–2.33	1.00			
Hypertension, yes/no	57/74	16/14	1.48	0.62–3.57	0.42			
Diabetes mellitus, yes/no	12/119	10/20	4.89	1.66–14.34	0.0016	2.29	0.75–7.00	0.15
Dyslipidemia, yes/no	29/102	7/23	1.070	0.35–2.92	1.00			
Cerebral hemorrhage (current or past), yes/no	5/126	1/29	0.87	0.018–8.21	1.00			
Cerebral infarction (current or past), yes/no	15/116	7/23	2.34	0.72–6.96	0.14			
Ischemic heart disease, yes/no	18/113	5/25	1.25	0.33–3.96	0.77			
Valvulitis (pre- and post-operative), yes/no	19/69 ^§^	4/17 ^§^	0.86	0.19–3.073	1.00			
Aortic stenosis (pre- and post-operative), yes/no	9/80 ^§^	1/20 ^§^	0.45	0.0097–3.57	0.68			
Aortic stenosis (pre-operative), yes/no	5/84 ^§^	1/20 ^§^	0.84	0.017–8.14	1.00			
Heart failure, yes/no	22/109	5/25	0.99	0.27–3.048	1.00			
Atrial fibrillation, yes/no	10/120 ^§^	5/25	2.38	0.59–8.49	0.16			

OGIB, obscure gastrointestinal bleeding; IVR, interventional radiology; OR, odds ratio; CI, confidence interval; SD, standard deviation; DOAC, direct oral anticoagulant; NSAIDs, non-steroidal anti-inflammatory drugs; PPI, proton pomp inhibitor; P-CAB, potassium-competitive acid blocker; WBC, white blood cells; Hb, hemoglobin; PT-INR, prothrombin time-international normalized ratio; BUN, blood urea nitrogen; Cr, creatinine; TP, total protein; Alb, albumin.

* Fisher‘s exact test

† Divided by median number

‡ The missing values are excluded due to unknown cases, transfer cases and bleeding at sites other than the small intestine

§ Data excluding missing value.

To determine whether vascular lesions were associated with any of the clinical features of OGIB, univariate analysis, followed by multivariate analysis, was performed and showed that diabetes mellitus was significantly associated (OR 3.45, 95% CI 1.23–9.70, *P* = 0.019) with vascular lesions (S7 Table).

Similar to the entire cohort, when examined only in the adult cohort, multivariate analysis after univariate analysis revealed that absence of vascular lesions (OR 4.89, 95% CI 1.85–12.90, *P* = 0.0014) were significantly associated with conservative treatment (S8 Table).

### Propensity score matched analysis

Following the 1:1 propensity score matching among those who underwent capsule endoscopy due to OGIB, 36 cases with active bleeding and 36 cases without active bleeding were assigned to each group (S1 Fig). As shown in S9 Table, the univariate analysis demonstrated that serum creatinine was significantly elevated in cases with active bleeding (OR 3.27, 95% CI 1.11–10.19, *P* = 0.029).

We were not able to match clinical features for lesion location in OGIB cases by age, sex, diabetes mellitus and heart failure. When considering treatment methods, 30 cases each were matched in two groups: the first with conservative treatment, and the second with invasive treatments such as endoscopic treatment, IVR, and surgery (S2 Fig). As shown in S10 Table, univariate analysis demonstrated that the absence of vascular lesions (OR 5.52, 95% CI 1.41–27.16, *P* = 0.010) was significantly associated with conservative treatment.

## Discussion

This was the first study to identify the comprehensive risk factors of clinical background and intestinal features for OGIB among patients who underwent capsule endoscopy. We demonstrated that vascular lesions of the small intestine and the use of DOAC were significantly associated with OGIB. We also showed that patients with OGIB without vascular lesions were acceptable for conservative treatment.

An association between portal hypertension and OGIB has been raised. Kunihara reported that exacerbation of esophageal varices and portal hypertensive gastropathy were independent predictors of portal hypertensive enteropathy exacerbation [14]. Clinically, portal hypertensive enteropathy could be a source of bleeding, including OGIB, in cirrhotic patients [15]. In our analyses, we found that some lifestyle-related diseases are associated with OGIB. Needless to say, there is a possibility that lifestyle-related diseases may be complicated by liver disease, but there have been no reports of a causal relationship between lifestyle-related diseases and OGIB or small intestinal bleeding. In previous reports, risk factors for upper gastrointestinal bleeding included lifestyle-related diseases and unhealthy habits, such as smoking [16, 17]. It is not surprising that these are also associated with small bowel bleeding.

Additionally, several drugs are also proven risk factors for upper gastrointestinal bleeding [16, 17]. There is a report on the relationship between gastrointestinal bleeding and DOAC; the American College of Cardiology, American Heart Association, and the Heart Rhythm Society joined to publish a guideline update stating that DOAC are considered superior to warfarin for preventing stroke and systemic embolism and were associated with lower risks of serious bleeding [18]. However, there has been only one report that accurately examined the relationship between DOAC usage in OGIB, including small intestinal bleeding. In particular, Silva suggested that DOAC usage was significantly associated with higher detection rates of P2 lesions on small bowel capsule endoscopy when adjusted for classical risk factors for mid-gastrointestinal bleeding [19]. Similarly, our analyses demonstrated that current DOAC usage was a risk factor for OGIB on multivariate and univariate analyses after propensity score matching. DOAC are of two types: the direct thrombin inhibitor; dabigatran, and the factor Xa inhibitors; rivaroxaban, apixaban, edoxaban, and betrixaban. They have increasingly been used as an alternative to warfarin and have been the agents of choice in most non-pregnant patients with atrial fibrillation and venous thromboembolism in the last decade [20, 21]. A previous study indicated that the risk of major gastrointestinal bleeding with DOAC did not differ from that with warfarin or low-molecular-weight heparin [22]. In contrast, according to the J-ROCKET AF study, rivaroxaban, which is a factor Xa inhibitor, tended to slightly increase gastrointestinal bleeding in patients aged 75 years and older, when compared to warfarin [23]. DOAC acts competitively on the active center of coagulation factors. Therefore, it is known that an anticoagulant effect is exerted immediately after administration, according to the blood concentration [24]. It is not surprising that DOAC tends to cause OGIB, including small bowel bleeding, as in our study.

Our analyses also showed that current PPI or P-cab use tended to be associated with the risk of OGIB through multivariate analysis. Omeprazole-induced gut dysbiosis promotes Akkermansia growth and inhibits Bifidobacterium growth, leading to a thinning of the mucus layer through a reduction in goblet cells in the small intestine [25]. Since compromised mucus protection can allow the entry of endotoxins and bacteria into blood vessels [26], Akkermansia, which is a genus in the phylum Verrucomicrobia [27], was considered to be involved in aspirin-induced injury caused by PPI [25]. According to past reports, P-CAB can be considered in the same way as PPI. Vonoprazan aggravated NSAIDs-induced small intestinal injury by reducing the population of L. johnsonii in the small intestine by suppressing gastric acid secretion [28]. These earlier reports support our results.

Our study also revealed that some lifestyle-related diseases were associated with OGIB. It has been reported that upper gastrointestinal bleeding was common in patients with coexisting hypertension and diabetes mellitus [29]. Diabetes was a significant risk factor for re-bleeding, with diabetic patients being three times more likely to re-bleed compared to non-diabetic patients [30]. It is known that in diabetic patients, abnormal angiogenesis is triggered by hypoxia due to impaired blood flow [31, 32]. Indeed, diabetes mellitus significantly coexisted in OGIB cases with vascular lesions. Our study could not demonstrate the association between OGIB and each lifestyle-related disease through the multivariate analyses. However, based on the results of our univariate analysis and past reports, it may be possible to consider an increased risk of OGIB in patients with coexisting lifestyle-related diseases.

Our study showed that low serum albumin at capsule endoscopy was a risk factor for active bleeding in multivariate analysis. Jiménez-Rosales et al. found that a low plasma albumin level was a risk factor for gastrointestinal bleeding and death in hospitalized patients [33, 34]. The level of albumin is lower in the high Glasgow-Blatchford Score (GBS) group than in the low GBS group, which was originally used to assess the need for acute management in patients with upper gastrointestinal bleeding, and predicts the need for intervention, and a high mortality rate, in patients with lower gastrointestinal bleeding [35, 36]. It is known that serum albumin levels can indirectly reflect nutritional status, such as dehydration, in patients with mucosal conditions [37]. Moreover, hypoalbuminemia has a heparin-like action and thus reduces coagulation ability [38]. Our results suggest that patients with hypoalbuminemia had more active gastrointestinal bleeding due to reduced coagulation.

Our study revealed that elevated creatinine at capsule endoscopy was also a risk factor for active bleeding among cases of OGIB from univariate analysis after propensity score matching. Patients with OGIB may also have prerenal deterioration of renal function due to continued gastrointestinal bleeding. In addition, hemodialysis was significantly associated with a 1.67-fold increased risk of GI bleeding compared with non-hemodialysis in multivariable analysis [39]. The pathophysiology of the bleeding tendency in patients with chronic kidney disease is multifactorial and includes platelet dysfunction and vessel wall damage [40].

The usefulness of several drugs for treatment of OGIB has been reported. Kim et al. found that the combined use of rebamipide with NSAIDs did not significantly increase the risk of OGIB compared to NSAIDs alone [41]. Baldassarre reported that Lactobacillus GG improves recovery in infants with blood in the stools in colitis [42]. In fact, in this study, there were multiple cases in which rebamipide, and intestinal regulators were initiated as conservative treatment for OGIB. Our results showed that the absence of vascular lesions was significantly associated with conservative treatment and the result persisted when propensity score matching was performed.

Double-balloon enteroscopy (DBE) is a relatively new endoscopic modality that allows for a more extensive evaluation of the small bowel and treatment of lesions previously inaccessible by conventional enteroscopy. The role of both capsule endoscopy and DBE in diagnosing and managing OGIB has been generally accepted. In fact, capsule endoscopy remains preferable than DBE in most circumstances when initiating the clinical evaluation of small bowel bleeding, since DBE creates patient distress and oral DBE runs a high risk of causing pancreatitis [43, 44]. In the case of OGIB, it is clinically important to first confirm the presence of vascular lesions by performing capsule endoscopy to identify cases that can resolve spontaneously without invasive treatment and to perform targeted DBE if necessary.

One of the limitations of our study is the retrospective study design. We therefore performed propensity score matching among patients with OGIB who underwent capsule endoscopy to reduce bias, although it should be noted that it only accounts for observed covariates. Since this was a retrospective study, it was not clear when each patient’s medication, including DOAC, was started. This is because most of the patients participating in this study were prescribed regular medications from other hospitals. However, the routine medications for each patient, including antithrombotic agents, appeared to have been taken at least 30 days before capsule endoscopy. The pathophysiological mechanisms may vary in pediatric and adult populations. Although we confirmed there was no noticeable difference in the results from adult data compared to the overall analysis, detailed analyses could not be performed among pediatric population data since the number of cases was small. Our study was limited to a single tertiary institution and, as a result, we could not examine the differences in treatment methods depending on the classification of vascular lesions due to the small number of cases. Thus, further studies are needed to assess generalizability. Moreover, our study does not reveal the post-treatment course of patients with small bowel bleeding and the prognosis could not be determined. In Japan, capsule endoscopy for the small intestine is not covered by insurance unless upper and lower gastrointestinal endoscopy is performed first. In addition, the cost of medical materials per test may be as high as 76,500 yen [45], so examinations cannot be performed unless we suspect small intestinal disease.

In conclusion, we demonstrated through our cohort study that DOAC was significantly associated with OGIB. OGIB cases with active bleeding had significantly lower serum albumin or elevated serum creatinine. In addition, while many OGIB cases had vascular lesions, cases without vascular lesions could be treated conservatively thereafter. Further larger studies are necessary to confirm our findings; however, the results of our study provide useful information for gastroenterologists and for interdisciplinary management of patients with OGIB.

## Supporting information

S1 Fig Flow chart of data analysis for comparison of clinical features in OGIB cases with and without active bleeding in patients who underwent capsule endoscopy, following propensity score matching with sex, age, diabetes mellitus and heart failure. OGIB, obscure gastrointestinal bleeding.
(TIF)

S2 Fig Flow chart of data analysis for comparison of clinical features in OGIB cases who received different treatments, following propensity score matching with sex, age, diabetes mellitus and heart failure. OGIB, obscure gastrointestinal bleeding; IVR, interventional radiology.
(TIF)

S1 Table Comparison of clinical features in cases that underwent capsule endoscopy, with and without vascular lesions, identified by univariate and multivariate analysis. (DOCX)

S2 Table Comparison of clinical features in adult patients who underwent capsule endoscopy, identified by univariate and multivariate analysis. (DOCX)

S3 Table Comparison of clinical features in pediatric patients who underwent capsule endoscopy, identified by univariate and multivariate analysis. (DOCX)

S4 Table Comparison of clinical features in adult cases of obscure gastrointestinal bleeding with and without active bleeding, identified by univariate and multivariate analysis. (DOCX)

S5 Table Comparison of clinical features according to lesion location in OGIB cases, identified by univariate analysis. (DOCX)

S6 Table Comparison of clinical features according to lesion location in adult OGIB cases, identified by univariate analysis. (DOCX)

S7 Table Comparison of clinical features in OGIB cases, with and without vascular lesions, identified by univariate and multivariate analysis. (DOCX)

S8 Table Comparison of clinical features of adult cases of obscure gastrointestinal bleeding receiving different treatments, identified by univariate and multivariate analysis. (DOCX)

S9 Table Comparison of clinical features of OGIB cases with and without active bleeding, following propensity score matching. (DOCX)

S10 Table Comparison of clinical features of OGIB cases receiving different treatments, following propensity score matching. (DOCX)

S1 File Raw data of clinical feature of capsule endoscopy. (XLSX)
