# Supplementary figures and images for: Clinical features of obscure gastrointestinal bleeding undergoing capsule endoscopy: A retrospective cohort study
Source: PLoS One. 2022 Mar 24;17(3):e0265903. doi: 10.1371/journal.pone.0265903 (PMC8947120; doi:10.1371/journal.pone.0265903)

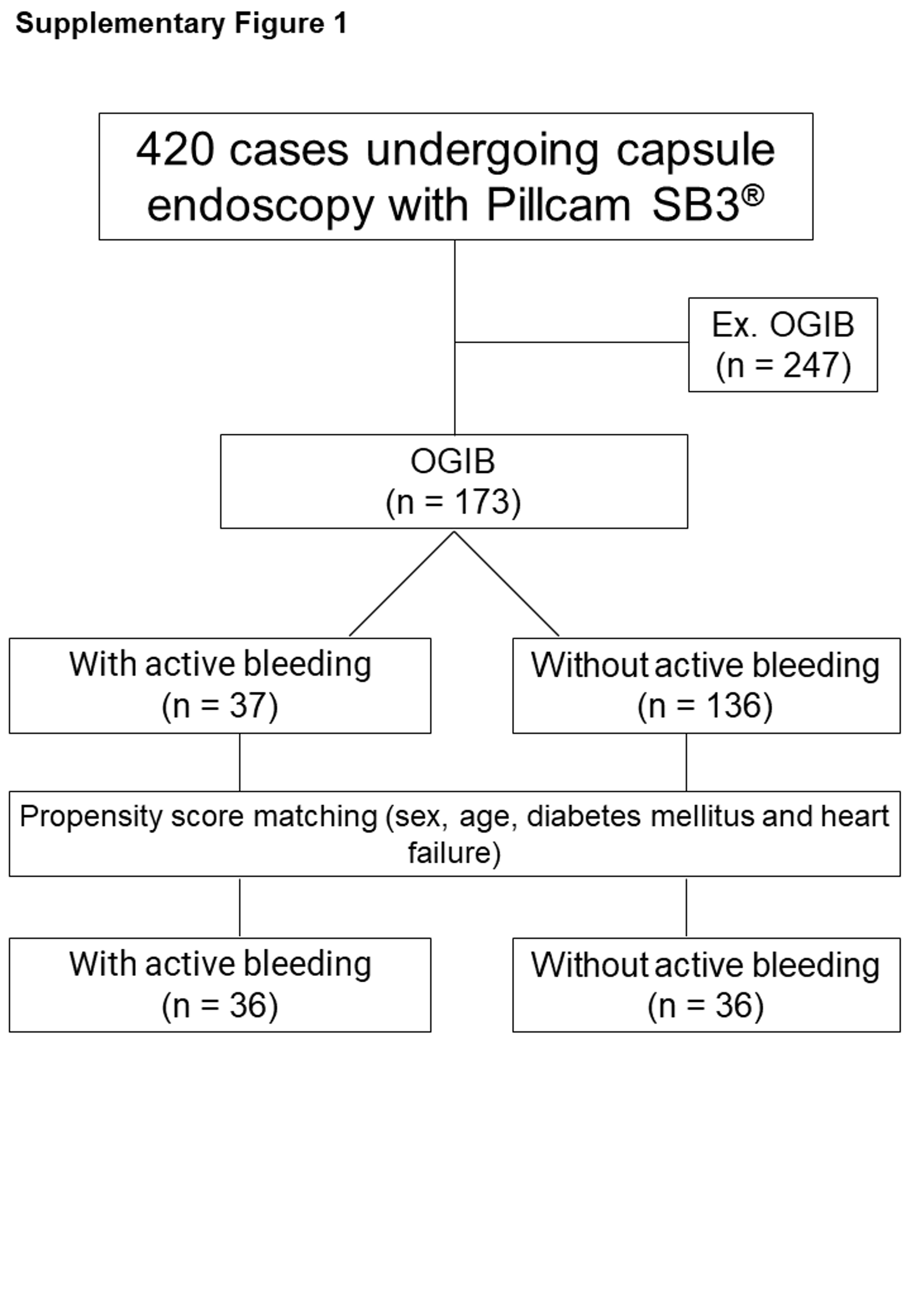

Supplement: S1 Fig — OGIB, obscure gastrointestinal bleeding. (TIF) [file pone.0265903.s001.tif]

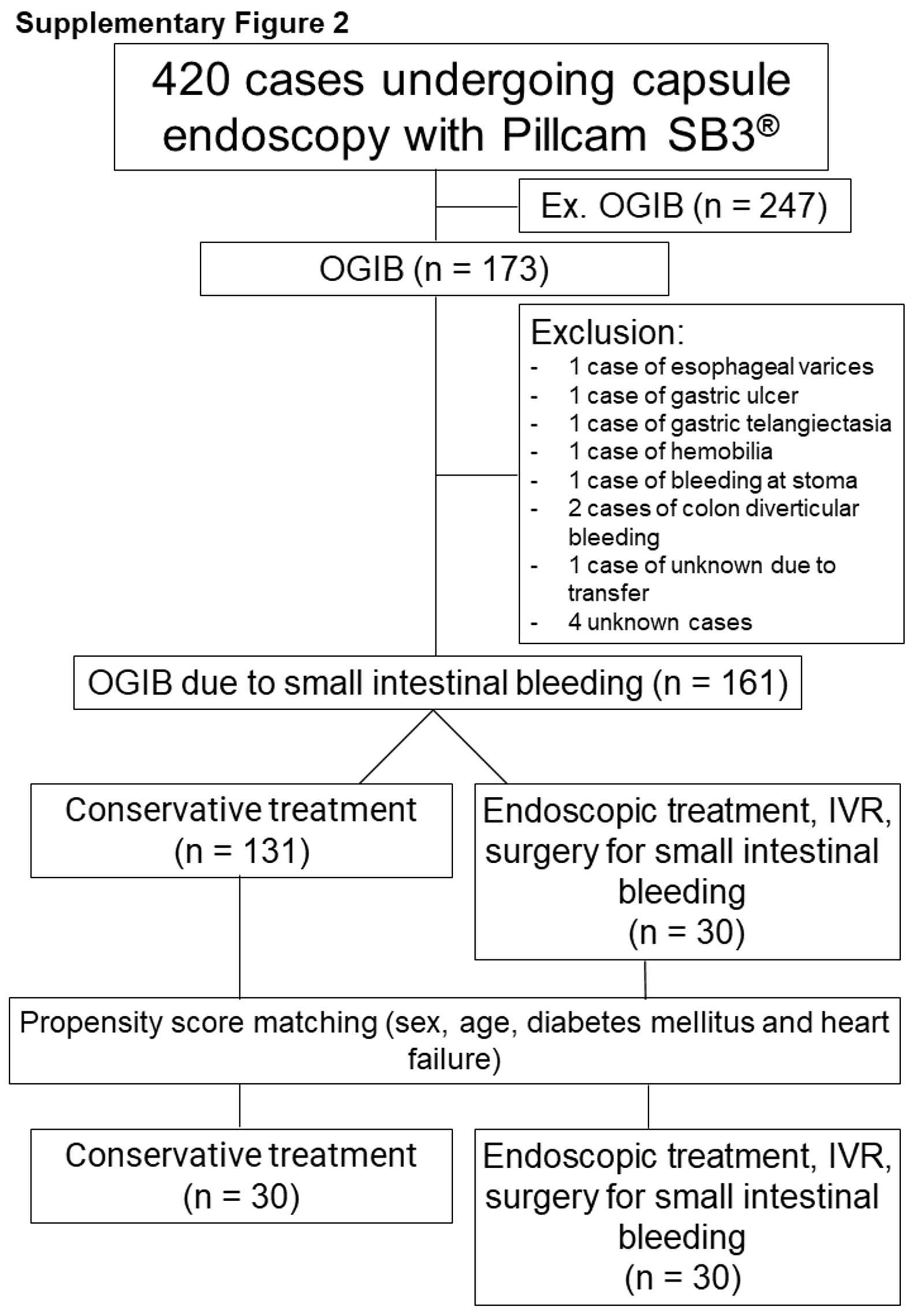

Supplement: S2 Fig — OGIB, obscure gastrointestinal bleeding; IVR, interventional radiology. (TIF) [file pone.0265903.s002.tif]
