# Supplementary material for: Clinical features of obscure gastrointestinal bleeding undergoing capsule endoscopy: A retrospective cohort study
Source: PLoS One. 2022 Mar 24;17(3):e0265903. doi: 10.1371/journal.pone.0265903 (PMC8947120; doi:10.1371/journal.pone.0265903)
Supplement: S1 Table — (DOCX) [file pone.0265903.s003.docx]

**S1 Table. Comparison of clinical features in cases that underwent capsule endoscopy, with and without vascular lesions, identified by univariate and multivariate analysis**

| **Factors** | **Cases that underwent capsule endoscopy **** | | **Univariate** | | | **Multivariate** | | |
| --- | --- | --- | --- | --- | --- | --- | --- | --- |
|  | With vascular lesions  (n = 36) | Without vascular lesions  (n = 383) | OR | 95% CI | *P ** | OR | 95% CI | *P ** |
| Age ≥ 59.15 years, yes/no (mean±SD) ^†^ | 29/7 (65.54±19.37) | 180/203 (50.33±22.67) | 4.66 | 1.94-12.91 | <0.0001 | 0.49 | 0.11-2.13 | 0.34 |
| Sex, male/female | 19/17 | 227/156 | 1.30 | 0.61-2.74 | 0.48 |  |  |  |
| Current or former smoker, yes/no | 10/23 *** | 133/215 *** | 0.70 | 0.60-2.31 | 0.45 |  |  |  |
| Current warfarin user, yes/no | 4/32 | 20/362 *** | 2.26 | 0.53-7.33 | 0.14 |  |  |  |
| Current DOAC user, yes/no | 3/33 | 16/366 *** | 2.075 | 0.37-7.80 | 0.22 |  |  |  |
| Current Aspirin user, yes/no | 4/32 | 30/352 *** | 1.47 | 0.35-4.55 | 0.52 |  |  |  |
| Current Thienopyridines user, yes/no | 1/35 | 14/368 *** | 0.75 | 0.017-5.23 | 1.00 |  |  |  |
| Current NSAIDs user, yes/no | 0/36 | 27/353 *** | 0.00 | 0.00-1.52 | 0.15 |  |  |  |
| Current probiotics user, yes/no | 1/35 | 57/323 *** | 0.16 | 0.0039-1.0077 | 0.043 | 3.97E-8 | 0.00-Inf. | 0.99 |
| Current PPI or P-CAB user, yes/no | 22/14 | 126/255 *** | 3.17 | 1.49-6.94 | 0.0016 | 0.82 | 0.23-2.84 | 0.75 |
| WBC ≥ 5,590.00/µL, yes/no (mean±SD) ^†^ | 13/23 (5,218.89±3,506.12) | 186/176 (6,206.77±2,871.61) *** | 0.54 | 0.026-0.30 | 0.12 |  |  |  |
| Hb ≥ 11.50 g/dL, yes/no (mean±SD) ^†^ | 4/32 (8.74±2.27) | 198/164 (11.59±2.83) *** | 0.10 | 0.75-2.71 | <0.0001 | 0.15 | 0.028-0.84 | 0.030 |
| Platelets ≥ 249.00/µL x10E3, yes/no (mean±SD) ^†^ | 8/28 (174.92±71.13) | 189/171 (262.96±120.19) *** | 0.26 | 0.099-0.60 | 0.00070 | 0.46 | 0.12-1.72 | 0.25 |
| PT-INR ≥ 1.040, yes/no (mean±SD) ^†^ | 22/13 (1.34±0.57) *** | 155/153 (1.096±0.27) *** | 1.67 | 0.77-3.74 | 0.21 |  |  |  |
| BUN ≥ 12.70 mg/dL, yes/no (mean±SD) ^†^ | 26/10 (24.43±19.55) | 175/182 (14.80±11.0093) *** | 2.70 | 1.22-6.46 | 0.0086 | 1.39 | 0.43-4.52 | 0.59 |
| Cr ≥ 0.76 mg/dL, yes/no (mean±SD) ^†^ | 20/16 (1.90±2.56) | 183/178 (0.96±1.054) *** | 1.22 | 0.58-2.60 | 0.60 |  |  |  |
| BUN/Cr ≥ 15.80, yes/no (mean±SD) ^†^ | 21/15 (20.72±12.38) | 177/183 (17.41±8.23) *** | 1.45 | 0.69-3.12 | 0.38 |  |  |  |
| TP ≥ 6.70 g/dL, yes/no (mean±SD) ^†^ | 8/24 (6.044±0.88) *** | 188/156 (6.57±1.050) *** | 0.28 | 0.10-0.66 | 0.0015 | 0.90 | 0.19-4.19 | 0.90 |
| Alb ≥ 3.80 g/dL, yes/no (mean±SD) ^†^ | 8/28 (3.092±0.78) | 186/155 (3.67±0.86) *** | 0.24 | 0.091-0.56 | 0.00033 | 1.37 | 0.32-5.82 | 0.67 |
| Hypertension, yes/no | 22/14 | 112/270 *** | 3.77 | 1.77-8.29 | 0.00025 | 3.59 | 1.00-12.90 | 0.050 |
| Diabetes mellitus, yes/no | 11/25 | 41/339 *** | 3.62 | 1.49-8.32 | 0.0022 | 1.36 | 0.34-5.45 | 0.66 |
| Dyslipidemia, yes/no | 8/28 | 60/321 *** | 1.53 | 0.57-3.65 | 0.34 |  |  |  |
| Cerebral hemorrhage (current or past), yes/no | 2/34 | 6/375 *** | 1.45 | 0.24-10.19 | 0.15 |  |  |  |
| Cerebral infarction (current or past), yes/no | 8/28 | 24/357 *** | 4.23 | 1.50-10.90 | 0.0033 | 1.52 | 0.37-6.21 | 0.56 |
| Ischemic heart disease, yes/no | 6/29 *** | 27/355 *** | 2.71 | 0.85-7.45 | 0.047 | 0.81 | 0.18-3.61 | 0.78 |
| Valvulitis (pre- and post-operative), yes/no | 9/18 *** | 24/171 *** | 3.54 | 1.25-9.48 | 0.0082 | 1.35 | 0.32-5.59 | 0.68 |
| Aortic stenosis (pre- and post-operative), yes/no | 3/24 *** | 12/185 *** | 1.92 | 0.32-7.85 | 0.40 |  |  |  |
| Aortic stenosis (pre-operative), yes/no | 2/25 *** | 8/188 *** | 1.87 | 0.18-10.16 | 0.35 |  |  |  |
| Heart failure, yes/no | 10/26 | 23/357 *** | 5.93 | 2.27-14.69 | 0.00015 | 5.00 | 1.080-23.10 | 0.039 |
| Atrial fibrillation, yes/no | 6/30 | 16/364 *** | 4.52 | 1.35-13.35 | 0.0074 | 0.70 | 0.11-4.32 | 0.70 |

OR, odds ratio; CI; confidence interval; SD, standard deviation; OGIB, obscure gastrointestinal bleeding; IBD, inflammatory bowel disease; DOAC, direct oral anticoagulant; NSAIDs, non-steroidal anti-inflammatory drugs; PPI, proton pomp inhibitor; P-CAB, potassium-competitive acid blocker; WBC, white blood cells; Hb, hemoglobin; PT-INR, prothrombin time-international normalized ratio; BUN, blood urea nitrogen; Cr, creatinine; TP, total protein; Alb, albumin.

* Fisher’s exact test; ** A missing value due to unknown legion was excluded; *** Data excluding missing value; † Divided by median number.
