# Supplementary material for: Clinical features of obscure gastrointestinal bleeding undergoing capsule endoscopy: A retrospective cohort study
Source: PLoS One. 2022 Mar 24;17(3):e0265903. doi: 10.1371/journal.pone.0265903 (PMC8947120; doi:10.1371/journal.pone.0265903)
Supplement: S2 Table — (DOCX) [file pone.0265903.s004.docx]

**S2 Table. Comparison of clinical features in adult patients who underwent capsule endoscopy, identified by univariate and multivariate analysis**

| **Factors** | **Reason for capsule endoscopy** | | **Univariate** | | | **Multivariate** | | |
| --- | --- | --- | --- | --- | --- | --- | --- | --- |
|  | OGIB  (n = 162) | Ex. OGIB ^‡^  (n = 207) | OR | 95% CI | *P ** | OR | 95% CI | *P ** |
| Age ≥ 62.56 years, yes/no (mean±SD) ^†^ | 108/54 (64.70±16.081) | 77/130 (50.59±19.038) | 3.36 | 2.15-5.32 | <0.0001 | 1.20 | 0.37-5.18 | 0.67 |
| Sex, male/female | 96/66 | 118/89 | 0.91 | 0.59-1.41 | 0.67 |  |  |  |
| Presence of erosion or ulcer, yes/no | 82/80 | 102/105 | 1.055 | 0.68-1.63 | 0.83 |  |  |  |
| Presence of vascular lesions, yes/no | 32/130 | 2/205 | 25.061 | 6.22-218.51 | <0.0001 | 11.00 | 1.040-115.00 | 0.046 |
| Current or former smoker, yes/no | 67/81 ^§^ | 76/107 ^§^ | 1.16 | 0.73-1.85 | 0.51 |  |  |  |
| Current warfarin user, yes/no | 19/143 | 5/201 ^§^ | 5.32 | 1.86-18.65 | 0.00044 | 4.17 | 0.58-29.80 | 0.16 |
| Current DOAC user, yes/no | 18/144 | 1/205 ^§^ | 25.47 | 3.93-1,067.27 | <0.0001 | 19.90 | 2.040-194.00 | 0.010 |
| Current Aspirin user, yes/no | 22/140 | 12/194 ^§^ | 2.53 | 1.16-5.82 | 0.017 | 1.82 | 0.42-7.88 | 0.42 |
| Current Thienopyridines user, yes/no | 8/154 | 7/199 ^§^ | 1.48 | 0.46-4.89 | 0.60 |  |  |  |
| Current NSAIDs user, yes/no | 11/151 | 16/188 ^§^ | 0.86 | 0.35-2.032 | 0.84 |  |  |  |
| Current probiotics user, yes/no | 19/141 ^§^ | 32/174 ^§^ | 0.73 | 0.38-1.40 | 0.36 |  |  |  |
| Current PPI or P-CAB user, yes/no | 83/79 | 65/140 ^§^ | 2.26 | 1.45-3.54 | 0.00018 | 0.43 | 0.17-1.090 | 0.074 |
| WBC ≥ 5,610.00/µL, yes/no (mean±SD) ^†^ | 68/92 (5,655.13±2,811.87) ^§^ | 106/83 (6,524.87±3,149.77) ^§^ | 0.58 | 0.37-0.91 | 0.014 | 0.37 | 0.15-0.94 | 0.036 |
| Platelets ≥ 235.00/µL x10E3, yes/no (mean±SD) ^†^ | 68/91 (217.68±114.17) | 106/82 (261.39±107.10) ^§^ | 0.58 | 0.37-0.91 | 0.013 | 1.86 | 0.71-4.91 | 0.21 |
| PT-INR ≥ 1.030, yes/no (mean±SD) ^†^ | 88/60 (1.21±0.44) ^§^ | 65/87 (1.034±0.13) ^§^ | 1.96 | 1.21-3.19 | 0.0040 | 0.85 | 0.35-2.10 | 0.73 |
| BUN ≥ 13.20 mg/dL, yes/no (mean±SD) ^†^ | 96/62 (20.59±17.058) ^§^ | 78/108 (13.18±5.87) ^§^ | 2.14 | 1.36-3.38 | 0.00055 | 1.33 | 0.50-3.52 | 0.57 |
| Cr ≥ 0.79 mg/dL, yes/no (mean±SD) ^†^ | 90/69 (1.40±1.85) ^§^ | 84/105 (0.87±0.64) ^§^ | 1.63 | 1.043-2.55 | 0.031 | 1.020 | 0.40-2.60 | 0.96 |
| BUN/Cr ≥ 15.84, yes/no (mean±SD) ^†^ | 84/75 (18.89±10.52) ^§^ | 90/98 (16.62±6.56) ^§^ | 1.22 | 0.78-1.90 | 0.39 |  |  |  |
| TP ≥ 6.60 g/dL, yes/no (mean±SD) ^†^ | 51/98 (6.054±1.00) ^§^ | 124/54 (6.77±1.0066) ^§^ | 0.23 | 0.14-0.37 | <0.0001 | 1.020 | 0.35-3.020 | 0.97 |
| Alb ≥ 3.80 g/dL, yes/no (mean±SD) ^†^ | 45/108 (3.25±0.77) ^§^ | 124/56 (3.89±0.85) ^§^ | 0.19 | 0.11-0.31 | <0.0001 | 0.29 | 0.090-0.90 | 0.032 |
| Hypertension, yes/no | 78/84 | 56/150 ^§^ | 2.48 | 1.57-3.94 | <0.0001 | 1.11 | 0.41-3.00 | 0.83 |
| Diabetes mellitus, yes/no | 23/138 ^§^ | 29/176 ^§^ | 1.011 | 0.53-1.90 | 1.00 |  |  |  |
| Dyslipidemia, yes/no | 39/122 ^§^ | 29/177 ^§^ | 1.95 | 1.11-3.46 | 0.015 | 0.93 | 0.28-3.040 | 0.90 |
| Cerebral hemorrhage (current or past), yes/no | 7/154 ^§^ | 1/205 ^§^ | 9.27 | 1.17-421.11 | 0.024 | 1.78E6 | 0.00-Inf. | 0.99 |
| Cerebral infarction (current or past), yes/no | 22/139 ^§^ | 10/196 ^§^ | 3.093 | 1.35-7.56 | 0.0045 | 1.12 | 0.23-5.45 | 0.89 |
| Ischemic heart disease, yes/no | 24/137 ^§^ | 9/197 ^§^ | 3.82 | 1.65-9.64 | 0.00071 | 2.80 | 0.57-13.80 | 0.21 |
| Valvulitis (pre- and post-operative), yes/no | 27/82 ^§^ | 6/88 ^§^ | 4.80 | 1.82-14.94 | 0.00046 | 3.26 | 0.59-17.90 | 0.17 |
| Aortic stenosis (pre- and post-operative), yes/no | 11/99 ^§^ | 4/91 ^§^ | 2.52 | 0.71-11.23 | 0.18 |  |  |  |
| Aortic stenosis (pre-operative), yes/no | 7/103 ^§^ | 3/91 ^§^ | 2.055 | 0.45-12.67 | 0.35 |  |  |  |
| Heart failure, yes/no | 29/132 ^§^ | 4/201 ^§^ | 10.97 | 3.73-43.90 | <0.0001 | 3.31 | 0.40-27.50 | 0.27 |
| Atrial fibrillation, yes/no | 18/143 ^§^ | 4/201 ^§^ | 7.00 | 12.016-26.12 | 0.00026 | 0.27 | 0.027-2.79 | 0.27 |

OR, odds ratio; CI, confidence interval; SD, standard deviation; OGIB, obscure gastrointestinal bleeding; IBD, inflammatory bowel disease; DOAC, direct oral anticoagulant; NSAIDs, non-steroidal anti-inflammatory drugs; PPI, proton pomp inhibitor; P-CAB, potassium-competitive acid blocker; WBC, white blood cells; Hb, hemoglobin; PT-INR, prothrombin time-international normalized ratio; BUN, blood urea nitrogen; Cr, creatinine; TP, total protein; Alb, albumin.

* Fisher’s exact test; † Divided by median number; ‡ Cases with inflammatory bowel disease, suspected small intestine tumor, diarrhea, stomach-ache and others without fecal occult blood or obvious bloody stool were included; § Data excluding missing value.
