# Supplementary material for: Clinical features of obscure gastrointestinal bleeding undergoing capsule endoscopy: A retrospective cohort study
Source: PLoS One. 2022 Mar 24;17(3):e0265903. doi: 10.1371/journal.pone.0265903 (PMC8947120; doi:10.1371/journal.pone.0265903)
Supplement: S3 Table — (DOCX) [file pone.0265903.s005.docx]

**S3 Table. Comparison of clinical features in pediatric patients who underwent capsule endoscopy, identified by univariate and multivariate analysis**

| **Factors** | **Reason for capsule endoscopy** | | **Univariate** | | |
| --- | --- | --- | --- | --- | --- |
|  | OGIB  (n = 11) | Ex. OGIB ^‡^  (n = 40) | OR | 95% CI | *P ** |
| Sex, male/female | 6/5 | 27/13 | 1.71 | 0.35-8.23 | 0.49 |
| Current warfarin user, yes/no | 0/11 | 0/40 |  |  |  |
| Current DOAC user, yes/no | 0/11 | 0/40 |  |  |  |
| Current Aspirin user, yes/no | 0/11 | 0/40 |  |  |  |
| Current Thienopyridines user, yes/no | 0/11 | 0/40 |  |  |  |
| Current NSAIDs user, yes/no | 0/11 | 0/40 |  |  |  |
| Current probiotics user, yes/no | 0/11 | 7/33 | 0.00 | 0.00-2.49 | 0.32 |
| Current PPI or P-CAB user, yes/no | 0/11 | 0/40 |  |  |  |
| WBC ≥ 5,520.00/µL, yes/no (mean±SD) ^†^ | 8/3 (6,826.36±3,014.26) | 17/21 (5,775.13±2,029.52) ^§^ | 3.22 | 0.64-21.74 | 0.17 |
| Platelets ≥ 340.50/µL x10E3, yes/no (mean±SD) ^†^ | 7/4 (343.45±159.97) | 18/21 (354.97±110.79) ^§^ | 2.013 | 0.43-10.98 | 0.50 |
| PT-INR ≥ 1.030, yes/no (mean±SD) ^†^ | 5/5 (1.22±0.40) ^§^ | 18/16 (1.10±0.093) ^§^ | 0.89 | 0.17-4.67 | 1.00 |
| BUN ≥ 9.60 mg/dL, yes/no (mean±SD) ^†^ | 7/4 (10.92±3.71) | 17/22 (8.81±3.30) ^§^ | 2.23 | 0.47-12.17 | 0.31 |
| Cr ≥ 0.55 mg/dL, yes/no (mean±SD) ^†^ | 3/8 (0.41±0.14) | 24/15 (0.60±0.17) ^§^ | 0.24 | 0.036-1.21 | 0.084 |
| BUN/Cr ≥ 15.62, yes/no (mean±SD) ^†^ | 9/2 (28.50±11.69) | 16/23 (14.93±5.63) ^§^ | 6.24 | 1.085-66.97 | 0.037 |
| TP ≥ 7.00 g/dL, yes/no (mean±SD) ^†^ | 5/6 (6.95±0.48) | 23/16 (7.14±0.76) ^§^ | 0.59 | 0.12-2.76 | 0.50 |
| Alb ≥ 3.90 g/dL, yes/no (mean±SD) ^†^ | 4/4 (3.43±0.87) ^§^ | 20/16 (3.82±0.80) ^§^ | 0.80 | 0.13-5.050 | 1.00 |
| Hypertension, yes/no | 0/11 | 0/40 |  |  |  |
| Diabetes mellitus, yes/no | 0/11 | 0/40 |  |  |  |
| Dyslipidemia, yes/no | 0/11 | 0/40 |  |  |  |
| Cerebral hemorrhage (current or past), yes/no | 0/11 | 0/40 |  |  |  |
| Cerebral infarction (current or past), yes/no | 0/11 | 0/40 |  |  |  |
| Ischemic heart disease, yes/no | 0/11 | 0/40 |  |  |  |
| Valvulitis (pre- and post-operative), yes/no | 0/7 ^§^ | 0/12 ^§^ |  |  |  |
| Aortic stenosis (pre- and post-operative), yes/no | 0/7 ^§^ | 0/12 ^§^ |  |  |  |
| Aortic stenosis (pre-operative), yes/no | 0/7 ^§^ | 0/12 ^§^ |  |  |  |
| Heart failure, yes/no | 0/11 | 0/40 |  |  |  |
| Atrial fibrillation, yes/no | 0/11 | 0/40 |  |  |  |

OR, odds ratio; CI, confidence interval; SD, standard deviation; OGIB, obscure gastrointestinal bleeding; IBD, inflammatory bowel disease; DOAC, direct oral anticoagulant; NSAIDs, non-steroidal anti-inflammatory drugs; PPI, proton pomp inhibitor; P-CAB, potassium-competitive acid blocker; WBC, white blood cells; Hb, hemoglobin; PT-INR, prothrombin time-international normalized ratio; BUN, blood urea nitrogen; Cr, creatinine; TP, total protein; Alb, albumin.

* Fisher’s exact test; † Divided by median number; ‡ Cases with inflammatory bowel disease, suspected small intestine tumor, diarrhea, stomach-ache and others without fecal occult blood or obvious bloody stool were included; § Data excluding missing value.
