# Supplementary material for: Clinical features of obscure gastrointestinal bleeding undergoing capsule endoscopy: A retrospective cohort study
Source: PLoS One. 2022 Mar 24;17(3):e0265903. doi: 10.1371/journal.pone.0265903 (PMC8947120; doi:10.1371/journal.pone.0265903)
Supplement: S4 Table — (DOCX) [file pone.0265903.s006.docx]

**S4 Table. Comparison of clinical features in adult cases of obscure gastrointestinal bleeding with and without active bleeding, identified by univariate and multivariate analysis**

| **Factors** | **Cases of OGIB** | | **Univariate** | | | **Multivariate** | | |
| --- | --- | --- | --- | --- | --- | --- | --- | --- |
|  | With active bleeding  (n = 36) | Without active bleeding  (n = 126) | OR | 95% CI | *P ** | OR | 95% CI | *P ** |
| Age ≥ 66.65 years, yes/no (mean±SD) ^†^ | 23/13 (68.29±14.42) | 58/68 (63.68±16.43) | 2.065 | 0.91-4.86 | 0.088 |  |  |  |
| Sex, male/female | 21/15 | 75/51 | 1.050 | 0.46-2.37 | 1.00 |  |  |  |
| Presence of erosion or ulcer, yes/no | 18/18 | 64/62 | 0.97 | 0.43-2.18 | 0.83 |  |  |  |
| Presence of vascular lesions, yes/no | 12/24 | 20/106 | 2.63 | 1.027-6.59 | 0.031 |  |  |  |
| Current or former smoker, yes/no | 17/16 ^‡^ | 50/65 ^‡^ | 1.38 | 0.59-3.24 | 0.43 |  |  |  |
| Current warfarin user, yes/no | 5/31 | 14/112 | 1.29 | 0.34-4.17 | 0.77 |  |  |  |
| Current DOAC user, yes/no | 4/32 | 14/112 | 1.00 | 0.22-3.49 | 1.00 |  |  |  |
| Current Aspirin user, yes/no | 7/29 | 15/111 | 1.78 | 0.56-5.18 | 0.27 |  |  |  |
| Current Thienopyridines user, yes/no | 2/34 | 6/120 | 1.18 | 0.11-6.96 | 1.00 |  |  |  |
| Current NSAIDs user, yes/no | 1/35 | 10/116 | 0.33 | 0.0074-2.49 | 0.46 |  |  |  |
| Current probiotics user, yes/no | 3/33 | 16/108 | 0.62 | 0.11-2.35 | 0.57 |  |  |  |
| Current PPI or P-CAB user, yes/no | 20/16 | 63/63 | 1.25 | 0.56-2.83 | 0.58 |  |  |  |
| WBC ≥ 5,055.00/µL, yes/no (mean±SD) ^†^ | 17/18 (5,447.43±2,654.27) ^‡^ | 62/62 (5,713.28±2,861.99) ^‡^ | 0.94 | 0.41-2.14 | 1.00 |  |  |  |
| Hb ≥ 9.00 g/dL, yes/no (mean±SD) ^†^ | 13/22 (8.65±2.49) ^‡^ | 68/57 (9.47±2.40) ^‡^ | 0.50 | 0.21-1.14 | 0.086 |  |  |  |
| Platelets ≥ 208.00/µL x10E3, yes/no (mean±SD) ^†^ | 12/22 (182.80±108.31) ^‡^ | 68/56 (227.45±114.28) ^‡^ | 0.45 | 0.19-1.051 | 0.053 |  |  |  |
| PT-INR ≥ 1.080, yes/no (mean±SD) ^†^ | 19/15 (1.29±0.58) ^‡^ | 56/59 (1.18±0.39) ^‡^ | 1.33 | 0.58-3.12 | 0.56 |  |  |  |
| BUN ≥ 15.050 mg/dL, yes/no (mean±SD) ^†^ | 23/12 (29.086±21.23) ^‡^ | 56/68 (18.20±14.93) ^‡^ | 2.32 | 1.0015-5.59 | 0.036 | 2.21 | 0.97-5.020 | 0.059 |
| Cr ≥ 0.83 mg/dL, yes/no (mean±SD) ^†^ | 22/12 (1.90±2.56) ^‡^ | 57/66 (0.96±1.054) ^‡^ | 2.11 | 0.91-5.13 | 0.081 |  |  |  |
| BUN/Cr ≥ 16.49, yes/no (mean±SD) ^†^ | 20/15 (21.047±12.88) ^‡^ | 59/65 (18.28±9.72) ^‡^ | 1.47 | 0.65-3.39 | 0.34 |  |  |  |
| TP ≥ 6.050 g/dL, yes/no (mean±SD) ^†^ | 13/21 (5.83±0.88) ^‡^ | 64/54 (6.12±1.022) ^‡^ | 0.55 | 0.23-1.28 | 0.17 |  |  |  |
| Alb ≥ 3.30 g/dL, yes/no (mean±SD) ^†^ | 11/23 (3.071±0.70) ^‡^ | 68/51 (3.31±0.79) ^‡^ | 0.36 | 0.14-0.85 | 0.012 | 0.40 | 0.17-0.91 | 0.029 |
| Hypertension, yes/no | 21/15 | 57/69 | 1.69 | 0.75-3.88 | 0.19 |  |  |  |
| Diabetes mellitus, yes/no | 9/26 ^‡^ | 14/112 | 2.75 | 0.94-7.72 | 0.052 |  |  |  |
| Dyslipidemia, yes/no | 12/23 ^‡^ | 27/99 | 1.90 | 0.76-4.62 | 0.12 |  |  |  |
| Cerebral hemorrhage (current or past), yes/no | 0/35 ^‡^ | 7/119 | 0.00 | 0.00-2.49 | 0.35 |  |  |  |
| Cerebral infarction (current or past), yes/no | 5/30 ^‡^ | 17/109 | 1.068 | 0.28-3.35 | 1.00 |  |  |  |
| Ischemic heart disease, yes/no | 8/27 ^‡^ | 16/110 | 2.027 | 0.68-5.67 | 0.18 |  |  |  |
| Valvulitis (pre- and post-operative), yes/no | 9/16 ^‡^ | 18/66 ^‡^ | 2.047 | 0.68-5.95 | 0.19 |  |  |  |
| Aortic stenosis (pre- and post-operative), yes/no | 4/22^‡^ | 7/77 ^‡^ | 1.99 | 0.39-8.69 | 0.29 |  |  |  |
| Aortic stenosis (pre-operative), yes/no | 3/23 ^‡^ | 4/80 ^‡^ | 2.58 | 0.35-16.48 | 0.35 |  |  |  |
| Heart failure, yes/no | 10/25 ^‡^ | 19/107 | 2.24 | 0.82-5.84 | 0.082 |  |  |  |
| Atrial fibrillation, yes/no | 5/31 | 13/112 ^‡^ | 1.39 | 0.36-4.56 | 0.56 |  |  |  |

OGIB, obscure gastrointestinal bleeding; OR, odds ratio; CI, confidence interval; SD, standard deviation; DOAC, direct oral anticoagulant; NSAIDs, non-steroidal anti-inflammatory drugs; PPI, proton pomp inhibitor; P-CAB, potassium-competitive acid blocker; WBC, white blood cells; Hb, hemoglobin; PT-INR, prothrombin time-international normalized ratio; BUN, blood urea nitrogen; Cr, creatinine; TP, total protein; Alb, albumin.

* Fisher’s exact test; † Divided by median number; ‡ Data excluding missing values.
