# Supplementary material for: Clinical features of obscure gastrointestinal bleeding undergoing capsule endoscopy: A retrospective cohort study
Source: PLoS One. 2022 Mar 24;17(3):e0265903. doi: 10.1371/journal.pone.0265903 (PMC8947120; doi:10.1371/journal.pone.0265903)
Supplement: S5 Table — (DOCX) [file pone.0265903.s007.docx]

**S5 Table. Comparison of clinical features according to lesion location in OGIB cases, identified by univariate analysis**

| **Factors** | **Location of bleeding in OGIB cases **** | | **Univariate** | | |
| --- | --- | --- | --- | --- | --- |
|  | Duodenum, Jejunum  (n = 36) | Ileum  (n = 29) | OR | 95% CI | *P ** |
| Age ≥ 66.38 years, yes/no (mean±SD) ^†^ | 17/19 (61.62±23.79) | 17/12 (61.62±17.13) | 0.64 | 0.21-1.89 | 0.46 |
| Sex, male/female | 23/13 | 15/14 | 0.61 | 0.20-1.84 | 0.45 |
| Presence of erosion or ulcer, yes/no | 24/12 | 20/9 | 0.90 | 0.27-2.90 | 1.00 |
| Presence of vascular lesions, yes/no | 11/25 | 9/20 | 0.98 | 0.30-3.25 | 1.00 |
| Current or former smoker, yes/no | 17/15 *** | 8/19 *** | 2.65 | 0.81-9.21 | 0.11 |
| Current warfarin user, yes/no | 5/31 | 2/27 | 2.15 | 0.32-24.35 | 0.45 |
| Current DOAC user, yes/no | 5/31 | 3/26 | 1.39 | 0.24-9.81 | 0.72 |
| Current Aspirin user, yes/no | 3/33 | 8/21 | 0.24 | 0.037-1.16 | 0.051 |
| Current Thienopyridines user, yes/no | 0/36 | 2/27 | 0.00 | 0.00-4.25 | 0.20 |
| Current NSAIDs user, yes/no | 0/36 | 1/28 | 0.00 | 0.00-31.42 | 0.45 |
| Current probiotics user, yes/no | 3/32 *** | 5/24 | 0.46 | 0.064-2.61 | 0.45 |
| Current PPI or P-CAB user, yes/no | 21/15 | 14/15 | 1.49 | 0.50-4.50 | 0.46 |
| WBC ≥ 5,080.00/µL, yes/no (mean±SD) ^†^ | 12/22 (5,194.00±2,084.13) *** | 17/12 (6,495.17±2,854.37) | 0.39 | 0.12-1.20 | 0.080 |
| Hb ≥ 9.050 g/dL, yes/no (mean±SD) ^†^ | 13/22 (8.56±2.47) *** | 18/11 (10.072±2.51) | 0.37 | 0.12-1.12 | 0.078 |
| Platelets ≥ 216.50/µL x10E3, yes/no (mean±SD) ^†^ | 13/21 (193.94±111.36) *** | 18/11 (242.00±103.48) | 0.38 | 0.12-1.17 | 0.079 |
| PT-INR ≥ 1.075, yes/no (mean±SD) ^†^ | 19/15 (1.26±0.41) *** | 15/14 (1.20±0.54) | 1.18 | 0.39-3.58 | 0.80 |
| BUN ≥ 14.80 mg/dL, yes/no (mean±SD) ^†^ | 20/15 (24.37±19.67) *** | 13/16 (20.36±18.82) | 1.62 | 0.54-4.97 | 0.45 |
| Cr ≥ 0.80 mg/dL, yes/no (mean±SD) ^†^ | 22/12 (1.84±2.28) *** | 13/16 (1.15±1.099) | 2.23 | 0.73-7.041 | 0.13 |
| BUN/Cr ≥ 16.83, yes/no (mean±SD) ^†^ | 16/19 (18.24±10.31) | 12/17 (19.069±10.75) | 1.19 | 0.40-3.63 | 0.80 |
| TP ≥ 6.20 g/dL, yes/no (mean±SD) ^†^ | 11/19 (5.92±0.90) *** | 13/15 (6.00±0.88) *** | 0.67 | 0.21-2.16 | 0.60 |
| Alb ≥ 3.30 g/dL, yes/no (mean±SD) ^†^ | 13/20 (3.10±0.64) *** | 13/15 (3.19±0.80) *** | 0.75 | 0.24-2.34 | 0.61 |
| Hypertension, yes/no | 19/17 | 13/16 | 1.37 | 0.46-4.12 | 0.62 |
| Diabetes mellitus, yes/no | 7/28 *** | 1/28 | 6.83 | 0.79-325.97 | 0.063 |
| Dyslipidemia, yes/no | 7/28 *** | 9/20 | 0.56 | 0.15-2.018 | 0.39 |
| Cerebral hemorrhage (current or past), yes/no | 3/32 *** | 1/28 | 2.59 | 0.20-142.53 | 0.62 |
| Cerebral infarction (current or past), yes/no | 5/30 *** | 3/26 | 1.44 | 0.25-10.14 | 0.72 |
| Ischemic heart disease, yes/no | 2/34 | 6/23 | 0.23 | 0.021-1.44 | 0.13 |
| Valvulitis (pre- and post-operative), yes/no | 5/19 *** | 4/13 *** | 0.86 | 0.15-5.21 | 1.00 |
| Aortic stenosis (pre- and post-operative), yes/no | 2/22 *** | 3/14 *** | 0.43 | 0.032-4.29 | 0.63 |
| Aortic stenosis (pre-operative), yes/no | 2/22 *** | 1/16 *** | 1.44 | 0.069-91.050 | 1.00 |
| Heart failure, yes/no | 5/30 *** | 6/23 | 0.64 | 0.14-2.89 | 0.53 |
| Atrial fibrillation, yes/no | 5/31 | 2/27 | 2.15 | 0.32-24.35 | 0.45 |

OGIB, obscure gastrointestinal bleeding; OR, odds ratio; CI, confidence interval; SD, standard deviation; IBD, inflammatory bowel disease; DOAC, direct oral anticoagulant; NSAIDs, non-steroidal anti-inflammatory drugs; PPI, proton pomp inhibitor; P-CAB, potassium-competitive acid blocker; WBC, white blood cells; Hb, hemoglobin; PT-INR, prothrombin time-international normalized ratio; BUN, blood urea nitrogen; Cr, creatinine; TP, total protein; Alb, albumin.

* Fisher’s exact test; ** Missing values due to no lesion in small intestine or cases with unknown localization were excluded; *** Data excluding missing value; † Divided by median number.
