# Supplementary material for: Clinical features of obscure gastrointestinal bleeding undergoing capsule endoscopy: A retrospective cohort study
Source: PLoS One. 2022 Mar 24;17(3):e0265903. doi: 10.1371/journal.pone.0265903 (PMC8947120; doi:10.1371/journal.pone.0265903)
Supplement: S6 Table — (DOCX) [file pone.0265903.s008.docx]

**S6 Table. Comparison of clinical features according to lesion location in adult OGIB cases, identified by univariate analysis**

| **Factors** | **Location of bleeding in OGIB cases **** | | **Univariate** | | |
| --- | --- | --- | --- | --- | --- |
|  | Duodenum, Jejunum  (n = 36) | Ileum  (n = 28) | OR | 95% CI | *P ** |
| Age ≥ 66.65 years, yes/no (mean±SD) ^†^ | 17/19 (61.62±23.79) | 16/12 (61.62±17.13) | 0.68 | 0.22-2.026 | 0.46 |
| Sex, male/female | 23/13 | 14/14 | 0.57 | 0.18-1.73 | 0.31 |
| Presence of erosion or ulcer, yes/no | 24/12 | 20/8 | 0.80 | 0.23-2.64 | 0.79 |
| Presence of vascular lesions, yes/no | 11/25 | 8/20 | 1.098 | 0.33-3.80 | 1.00 |
| Current or former smoker, yes/no | 17/15 *** | 8/18 *** | 2.51 | 0.76-8.78 | 0.11 |
| Current warfarin user, yes/no | 5/31 | 2/26 | 2.074 | 0.31-23.49 | 0.45 |
| Current DOAC user, yes/no | 5/31 | 3/25 | 1.34 | 0.24-9.46 | 1.00 |
| Current Aspirin user, yes/no | 3/33 | 8/20 | 0.23 | 0.036-1.11 | 0.047 |
| Current Thienopyridines user, yes/no | 0/36 | 2/26 | 0.00 | 0.00-4.10 | 0.19 |
| Current NSAIDs user, yes/no | 0/36 | 1/27 | 0.00 | 0.00-30.33 | 0.44 |
| Current probiotics user, yes/no | 3/32 *** | 5/23 | 0.44 | 0.062-2.51 | 0.45 |
| Current PPI or P-CAB user, yes/no | 21/15 | 14/14 | 1.39 | 0.46-4.24 | 0.62 |
| WBC ≥ 5,055.00/µL, yes/no (mean±SD) ^†^ | 12/22 (5,194.00±2,084.13) *** | 16/12 (6,494.29±2,906.74) | 0.42 | 0.13-1.28 | 0.12 |
| Hb ≥ 9.00 g/dL, yes/no (mean±SD) ^†^ | 13/22 (8.56±2.47) *** | 17/11 (10.032±2.55) | 0.39 | 0.12-1.19 | 0.073 |
| Platelets ≥ 208.00/µL x10E3, yes/no (mean±SD) ^†^ | 13/21 (193.94±111.36) *** | 18/10 (240.79±105.17) | 0.35 | 0.11-1.088 | 0.081 |
| PT-INR ≥ 1.080, yes/no (mean±SD) ^†^ | 19/15 (1.26±0.41) *** | 15/13 (1.21±0.55) | 1.096 | 0.36-3.37 | 1.00 |
| BUN ≥ 15.050 mg/dL, yes/no (mean±SD) ^†^ | 20/15 (24.37±19.67) *** | 11/17 (20.63±19.11) | 2.037 | 0.67-6.42 | 0.21 |
| Cr ≥ 0.80 mg/dL, yes/no (mean±SD) ^†^ | 22/12 (1.84±2.28) *** | 12/16 (1.18±1.11) | 2.41 | 0.78-7.74 | 0.12 |
| BUN/Cr ≥ 16.49, yes/no (mean±SD) ^†^ | 16/19 (18.24±10.31) *** | 12/16 (18.0053±9.27) | 0.74 | 0.24-2.21 | 0.62 |
| TP ≥ 6.050 g/dL, yes/no (mean±SD) ^†^ | 11/19 (5.92±0.90) *** | 13/14 (5.99±0.89) *** | 0.63 | 0.19-2.039 | 0.43 |
| Alb ≥ 3.30 g/dL, yes/no (mean±SD) ^†^ | 13/20 (3.10±0.64) *** | 12/15 (3.14±0.78) *** | 0.82 | 0.26-2.58 | 0.79 |
| Hypertension, yes/no | 19/17 | 13/15 | 1.28 | 0.43-3.89 | 0.80 |
| Diabetes mellitus, yes/no | 7/28 *** | 1/27 | 6.58 | 0.76-314.75 | 0.066 |
| Dyslipidemia, yes/no | 7/28 *** | 9/19 | 0.53 | 0.14-1.93 | 0.38 |
| Cerebral hemorrhage (current or past), yes/no | 3/32 *** | 1/27 | 2.50 | 0.19-137.62 | 0.62 |
| Cerebral infarction (current or past), yes/no | 5/30 *** | 3/25 | 1.38 | 0.24-9.78 | 0.72 |
| Ischemic heart disease, yes/no | 2/34 | 6/22 | 0.22 | 0.020-1.38 | 0.12 |
| Valvulitis (pre- and post-operative), yes/no | 5/19 *** | 4/13 *** | 0.86 | 0.15-5.21 | 1.00 |
| Aortic stenosis (pre- and post-operative), yes/no | 2/22 *** | 3/14 *** | 0.43 | 0.032-4.29 | 0.63 |
| Aortic stenosis (pre-operative), yes/no | 2/22 *** | 1/16 *** | 1.44 | 0.069-91.050 | 1.00 |
| Heart failure, yes/no | 5/30 *** | 6/22 | 0.62 | 0.13-2.77 | 0.52 |
| Atrial fibrillation, yes/no | 5/31 | 2/26 | 2.074 | 0.31-23.49 | 0.45 |

OGIB, obscure gastrointestinal bleeding; OR, odds ratio; CI, confidence interval; SD, standard deviation; IBD, inflammatory bowel disease; DOAC, direct oral anticoagulant; NSAIDs, non-steroidal anti-inflammatory drugs; PPI, proton pomp inhibitor; P-CAB, potassium-competitive acid blocker; WBC, white blood cells; Hb, hemoglobin; PT-INR, prothrombin time-international normalized ratio; BUN, blood urea nitrogen; Cr, creatinine; TP, total protein; Alb, albumin.

* Fisher’s exact test; ** Missing values due to no lesion in small intestine or cases with unknown localization were excluded; *** Data excluding missing value; † Divided by median number.
