# Supplementary material for: Clinical features of obscure gastrointestinal bleeding undergoing capsule endoscopy: A retrospective cohort study
Source: PLoS One. 2022 Mar 24;17(3):e0265903. doi: 10.1371/journal.pone.0265903 (PMC8947120; doi:10.1371/journal.pone.0265903)
Supplement: S7 Table — (DOCX) [file pone.0265903.s009.docx]

**S7 Table. Comparison of clinical features in OGIB cases, with and without vascular lesions, identified by univariate and multivariate analysis**

| **Factors** | **Cases of OGIB** | | **Univariate** | | | **Multivariate** | | |
| --- | --- | --- | --- | --- | --- | --- | --- | --- |
|  | With vascular lesions  (n = 34) | Without vascular lesions  (n = 139) | OR | 95% CI | *P ** | OR | 95% CI | *P ** |
| Age ≥ 66.38 years, yes/no (mean±SD) ^†^ | 24/10 (67.37±18.29) | 63/76 (59.74±20.83) | 2.88 | 1.22-7.28 | 0.012 | 1.56 | 0.62-3.94 | 0.35 |
| Sex, male/female | 19/15 | 83/56 | 1.17 | 0.51-2.66 | 0.70 |  |  |  |
| Current or former smoker, yes/no | 10/21 ** | 57/71 ** | 0.60 | 0.23-1.45 | 0.23 |  |  |  |
| Current warfarin user, yes/no | 4/30 | 15/124 | 1.10 | 0.25-3.80 | 1.00 |  |  |  |
| Current DOAC user, yes/no | 3/31 | 15/124 | 0.80 | 0.14-3.092 | 1.00 |  |  |  |
| Current Aspirin user, yes/no | 4/30 | 18/121 | 0.90 | 0.21-3.0080 | 1.00 |  |  |  |
| Current Thienopyridines user, yes/no | 1/33 | 7/132 | 0.57 | 0.012-4.72 | 1.00 |  |  |  |
| Current NSAIDs user, yes/no | 0/34 | 11/128 | 0.00 | 0.00-1.59 | 0.13 |  |  |  |
| Current probiotics user, yes/no | 1/33 | 18/119 ** | 0.20 | 0.0047-1.37 | 0.13 |  |  |  |
| Current PPI or P-CAB user, yes/no | 20/14 | 63/76 | 1.72 | 0.76-4.0011 | 0.18 |  |  |  |
| WBC ≥ 5,080.00/µL, yes/no (mean±SD) ^†^ | 13/21 (5,105.88±3,496.59) | 73/63 (5,885.47±2,631.97) ** | 0.54 | 0.23-1.23 | 0.13 |  |  |  |
| Hb ≥ 9.050 g/dL, yes/no (mean±SD) ^†^ | 11/23 (8.60±2.25) | 74/63 (9.50±2.46) ** | 0.41 | 0.17-0.95 | 0.034 | 0.60 | 0.25-1.42 | 0.25 |
| Platelets ≥ 216.50/µL x10E3, yes/no (mean±SD) ^†^ | 11/23 (174.65±71.094) | 74/61 (238.46±127.58) ** | 0.40 | 0.16-0.93 | 0.022 | 0.66 | 0.27-1.60 | 0.35 |
| PT-INR ≥ 1.075, yes/no (mean±SD) ^†^ | 21/13 (1.35±0.57) | 59/66 (1.17±0.38) ** | 1.80 | 0.78-4.29 | 0.18 |  |  |  |
| BUN ≥ 14.80 mg/dL, yes/no (mean±SD) ^†^ | 21/13 (25.14±19.86) | 65/71 (18.68±15.62) ** | 1.76 | 0.77-4.16 | 0.18 |  |  |  |
| Cr ≥ 0.80 mg/dL, yes/no (mean±SD) ^†^ | 20/14 (1.98±2.61) | 67/67 (1.18±1.51) ** | 1.43 | 0.63-3.33 | 0.44 |  |  |  |
| BUN/Cr ≥ 16.83, yes/no (mean±SD) ^†^ | 19/15 (20.64±12.48) | 65/71 (19.23±10.40) ** | 1.38 | 0.61-3.19 | 0.45 |  |  |  |
| TP ≥ 6.20 g/dL, yes/no (mean±SD) ^†^ | 14/16 (5.87±0.90) ** | 66/64 (6.19±1.013) ** | 0.85 | 0.35-2.029 | 0.84 |  |  |  |
| Alb ≥ 3.30 g/dL, yes/no (mean±SD) ^†^ | 13/21 (3.074±0.69) | 70/57 (3.31±0.79) ** | 0.51 | 0.21-1.17 | 0.086 |  |  |  |
| Hypertension, yes/no | 20/14 | 58/81 | 1.99 | 0.87-4.64 | 0.085 |  |  |  |
| Diabetes mellitus, yes/no | 11/23 | 12/126 ** | 4.96 | 1.76-14.020 | 0.00097 | 3.45 | 1.23-9.70 | 0.019 |
| Dyslipidemia, yes/no | 7/27 | 32/106 ** | 0.86 | 0.29-2.27 | 0.82 |  |  |  |
| Cerebral hemorrhage (current or past), yes/no | 2/32 | 5/133 ** | 1.66 | 0.15-10.70 | 0.63 |  |  |  |
| Cerebral infarction (current or past), yes/no | 8/26 | 14/124 ** | 2.71 | 0.89-7.79 | 0.047 | 1.48 | 0.50-4.43 | 0.48 |
| Ischemic heart disease, yes/no | 6/27 ** | 18/121 | 1.49 | 0.44-4.41 | 0.41 |  |  |  |
| Valvulitis (pre- and post-operative), yes/no | 9/16 ** | 18/73 ** | 2.26 | 0.75-6.55 | 0.11 |  |  |  |
| Aortic stenosis (pre- and post-operative), yes/no | 3/22 ** | 8/84 ** | 1.43 | 0.23-6.61 | 0.70 |  |  |  |
| Aortic stenosis (pre-operative), yes/no | 2/23 ** | 5/87 ** | 1.51 | 0.14-9.96 | 0.64 |  |  |  |
| Heart failure, yes/no | 10/24 | 19/119 ** | 2.59 | 0.95-6.77 | 0.040 | 1.63 | 0.58-4.54 | 0.35 |
| Atrial fibrillation, yes/no | 6/28 | 12/126 ** | 2.24 | 0.63-7.14 | 0.21 |  |  |  |

OGIB, obscure gastrointestinal bleeding; OR, odds ratio; CI, confidence interval; SD, standard deviation; IBD, inflammatory bowel disease; DOAC, direct oral anticoagulant; NSAIDs, non-steroidal anti-inflammatory drugs; PPI, proton pomp inhibitor; P-CAB, potassium-competitive acid blocker; WBC, white blood cells; Hb, hemoglobin; PT-INR, prothrombin time-international normalized ratio; BUN, blood urea nitrogen; Cr, creatinine; TP, total protein; Alb, albumin.

* Fisher’s exact test; ** Data excluding missing value; † Divided by median number.
