# Supplementary material for: Clinical features of obscure gastrointestinal bleeding undergoing capsule endoscopy: A retrospective cohort study
Source: PLoS One. 2022 Mar 24;17(3):e0265903. doi: 10.1371/journal.pone.0265903 (PMC8947120; doi:10.1371/journal.pone.0265903)
Supplement: S8 Table — (DOCX) [file pone.0265903.s010.docx]

**S8 Table. Comparison of clinical features of adult cases of obscure gastrointestinal bleeding receiving different treatments, identified by univariate and multivariate analysis**

| **Factors** | **Cases of OGIB** ^‡^ | | **Univariate** | | | **Multivariate** | | |
| --- | --- | --- | --- | --- | --- | --- | --- | --- |
|  | Conservative treatment  (n = 122) | Endoscopic treatment,  IVR, surgery  (n = 29) | OR | 95% CI | *P ** | OR | 95% CI | *P ** |
| Age ≥ 66.65 years, yes/no (mean±SD) ^†^ | 58/64 (64.45±15.50) | 18/11 (65.015±19.60) | 1.80 | 0.73-4.59 | 0.22 |  |  |  |
| Sex, male/female | 68/54 | 21/8 | 0.48 | 0.17-1.24 | 0.14 |  |  |  |
| Presence of erosion or ulcer, yes/no | 66/56 | 11/18 | 0.52 | 0.20-1.28 | 0.15 |  |  |  |
| Presence of vascular lesions, yes/no | 15/107 | 14/15 | 6.54 | 2.42-18.067 | <0.0001 | 4.89 | 1.85-12.90 | 0.0014 |
| Current or former smoker, yes/no | 52/63 ^§^ | 11/14 ^§^ | 0.95 | 0.36-2.48 | 1.00 |  |  |  |
| Current warfarin user, yes/no | 13/109 | 4/25 | 1.34 | 0.29-4.84 | 0.74 |  |  |  |
| Current DOAC user, yes/no | 14/108 | 1/28 | 0.28 | 0.0063-1.98 | 0.31 |  |  |  |
| Current Aspirin user, yes/no | 19/103 | 3/26 | 0.63 | 0.11-2.38 | 0.57 |  |  |  |
| Current Thienopyridines user, yes/no | 8/114 | 0/29 | 0 | 0.00-2.46 | 0.35 |  |  |  |
| Current NSAIDs user, yes/no | 11/111 | 0/29 | 0.00 | 0.00-1.64 | 0.12 |  |  |  |
| Current probiotics user, yes/no | 16/104 | 1/28 | 0.23 | 0.0054-1.64 | 0.20 |  |  |  |
| Current PPI or P-CAB user, yes/no | 59/63 | 15/14 | 1.14 | 0.47-2.80 | 0.84 |  |  |  |
| WBC ≥ 5,055.00/µL, yes/no (mean±SD) ^†^ | 63/57 (5,846.033±3,026.21) ^§^ | 11/18 (5,030.35±1,998.59) | 0.56 | 0.22-1.36 | 0.21 |  |  |  |
| Hb ≥ 9.00 g/dL, yes/no (mean±SD) ^†^ | 69/52 (9.52±2.44) ^§^ | 8/21 (8.40±2.44) | 0.29 | 0.10-0.75 | 0.0066 | 0.47 | 0.17-1.27 | 0.14 |
| Platelets ≥ 208.00/µL x10E3, yes/no (mean±SD) ^†^ | 69/51 (233.68±119.35) ^§^ | 9/19 (168.83±82.75) ^§^ | 0.35 | 0.13-0.90 | 0.020 | 0.51 | 0.20-1.35 | 0.18 |
| PT-INR ≥ 1.080, yes/no (mean±SD) ^†^ | 55/55 (1.19±0.40) ^§^ | 15/14 (1.24±0.58) | 1.071 | 0.44-2.65 | 1.00 |  |  |  |
| BUN ≥ 15.050 mg/dL, yes/no (mean±SD) ^†^ | 55/65 (19.13±15.79) ^§^ | 20/9 (28.32±21.85) | 2.61 | 1.034-7.071 | 0.038 | 1.22 | 0.44-3.40 | 0.70 |
| Cr ≥ 0.83 mg/dL, yes/no (mean±SD) ^†^ | 57/62 (1.2±1.37) ^§^ | 19/9 (2.38±3.17) ^§^ | 2.28 | 0.90-6.22 | 0.062 |  |  |  |
| BUN/Cr ≥ 16.49, yes/no (mean±SD) ^†^ | 59/61 (18.51±8.76) ^§^ | 15/14 (20.92±15.91) | 1.11 | 0.45-2.72 | 0.84 |  |  |  |
| TP ≥ 6.050 g/dL, yes/no (mean±SD) ^†^ | 59/54 (6.12±0.99) ^§^ | 11/15 (5.78±1.034) ^§^ | 0.67 | 0.26-1.73 | 0.39 |  |  |  |
| Alb ≥ 3.30 g/dL, yes/no (mean±SD) ^†^ | 59/55 (3.26±0.77) ^§^ | 14/15 (3.19±0.79) | 0.87 | 0.35-2.13 | 0.84 |  |  |  |
| Hypertension, yes/no | 57/65 | 16/13 | 1.40 | 0.58-3.46 | 0.54 |  |  |  |
| Diabetes mellitus, yes/no | 12/110 | 10/19 | 4.76 | 1.60-14.062 | 0.0021 | 1.93 | 0.61-6.14 | 0.26 |
| Dyslipidemia, yes/no | 29/93 | 7/22 | 1.020 | 0.33-2.80 | 1.00 |  |  |  |
| Cerebral hemorrhage (current or past), yes/no | 5/117 | 1/28 | 0.84 | 0.017-7.92 | 1.00 |  |  |  |
| Cerebral infarction (current or past), yes/no | 15/107 | 7/22 | 2.26 | 0.69-6.76 | 0.14 |  |  |  |
| Ischemic heart disease, yes/no | 18/104 | 5/24 | 1.20 | 0.32-3.82 | 0.78 |  |  |  |
| Valvulitis (pre- and post-operative), yes/no | 19/63 ^§^ | 4/16^§^ | 0.83 | 0.18-3.018 | 1.00 |  |  |  |
| Aortic stenosis (pre- and post-operative), yes/no | 9/74 ^§^ | 1/19 ^§^ | 0.44 | 0.0094-3.50 | 0.68 |  |  |  |
| Aortic stenosis (pre-operative), yes/no | 5/78 ^§^ | 1/19 ^§^ | 0.82 | 0.017-8.00 | 1.00 |  |  |  |
| Heart failure, yes/no | 22/100 | 5/24 | 0.95 | 0.25-2.93 | 1.00 |  |  |  |
| Atrial fibrillation, yes/no | 10/111 ^§^ | 5/24 | 2.30 | 0.56-8.23 | 0.17 |  |  |  |

OGIB, obscure gastrointestinal bleeding; IVR, interventional radiology; OR, odds ratio; CI, confidence interval; SD, standard deviation; DOAC, direct oral anticoagulant; NSAIDs, non-steroidal anti-inflammatory drugs; PPI, proton pomp inhibitor; P-CAB, potassium-competitive acid blocker; WBC, white blood cells; Hb, hemoglobin; PT-INR, prothrombin time-international normalized ratio; BUN, blood urea nitrogen; Cr, creatinine; TP, total protein; Alb, albumin.

* Fisher’s exact test; † Divided by median number; ‡ The missing values are excluded due to unknown cases, transfer cases and bleeding at sites other than the small intestine; § Data excluding missing value.
