# Supplementary material for: Clinical features of obscure gastrointestinal bleeding undergoing capsule endoscopy: A retrospective cohort study
Source: PLoS One. 2022 Mar 24;17(3):e0265903. doi: 10.1371/journal.pone.0265903 (PMC8947120; doi:10.1371/journal.pone.0265903)
Supplement: S9 Table — (DOCX) [file pone.0265903.s011.docx]

**S9 Table. Comparison of clinical features of OGIB cases with and without active bleeding, following propensity score matching**

| **Factors** | **Cases of OGIB** | | **Univariate** | | |
| --- | --- | --- | --- | --- | --- |
|  | With active bleeding  (n = 36) | Without active bleeding  (n = 36) | OR | 95% CI | *P* * |
| Presence of erosion or ulcer, yes/no | 17/19 | 15/21 | 1.25 | 0.45-3.52 | 0.81 |
| Presence of vascular lesions, yes/no | 12/24 | 9/27 | 1.49 | 0.48-4.79 | 0.61 |
| Current or former smoker, yes/no | 17/17 ** | 10/20 ** | 1.98 | 0.65-6.27 | 0.21 |
| Current warfarin user, yes/no | 5/31 | 3/33 | 1.29 | 0.25-7.14 | 0.71 |
| Current DOAC user, yes/no | 4/32 | 7/29 | 0.52 | 0.10-2.31 | 0.51 |
| Current Aspirin user, yes/no | 7/29 | 3/33 | 2.62 | 0.54-17.15 | 0.31 |
| Current Thienopyridines user, yes/no | 2/34 | 1/35 | 2.039 | 0.10-124.78 | 1.00 |
| Current NSAIDs user, yes/no | 1/35 | 0/36 | Inf. | 0.026-Inf. | 1.00 |
| Current probiotics user, yes/no | 3/33 | 6/28 ** | 0.43 | 0.064-2.23 | 0.30 |
| Current PPI or P-CAB user, yes/no | 19/17 | 20/16 | 0.90 | 0.32-2.50 | 1.00 |
| WBC ≥ 5,080/µL, yes/no ^†^ | 17/19 | 18/17 ** | 0.85 | 0.30-2.37 | 0.81 |
| Hb ≥ 9.050 g/dL, yes/no ^†^ | 12/24 | 16/20 | 0.63 | 0.22-1.80 | 0.47 |
| Platelets ≥ 216.50/µL x10E3, yes/no ^†^ | 12/23 ** | 12/24 | 1.043 | 0.35-3.13 | 1.00 |
| PT-INR ≥ 1.075, yes/no ^†^ | 20/15 ** | 21/12 ** | 0.76 | 0.26-2.25 | 0.63 |
| BUN ≥ 14.80 mg/dL, yes/no ^†^ | 24/12 | 21/14 ** | 1.33 | 0.45-3.93 | 0.63 |
| Cr ≥ 0.80 mg/dL, yes/no ^†^ | 25/10 ** | 15/20 ** | 3.27 | 1.11-10.19 | 0.029 |
| BUN/Cr ≥ 16.83, yes/no ^†^ | 21/15 | 18/17 ** | 1.32 | 0.47-3.74 | 0.64 |
| TP ≥ 6.20 g/dL, yes/no ^†^ | 13/22 ** | 16/17 ** | 0.63 | 0.21-1.84 | 0.46 |
| Alb ≥ 3.30 g/dL, yes/no ^†^ | 11/24 ** | 16/18 ** | 0.52 | 0.17-1.53 | 0.22 |
| Hypertension, yes/no | 20/16 | 17/19 | 1.39 | 0.50-3.91 | 0.64 |
| Dyslipidemia, yes/no | 12/24 | 9/27 | 1.49 | 0.48-4.79 | 0.61 |
| Cerebral hemorrhage (current or past), yes/no | 0/36 | 2/34 | 0.00 | 0.00-5.30 | 0.49 |
| Cerebral infarction (current or past), yes/no | 5/31 | 8/28 | 0.57 | 0.13-2.25 | 0.54 |
| Ischemic heart disease, yes/no | 8/27 ** | 8/28 | 1.037 | 0.29-3.68 | 1.00 |
| Valvulitis (pre- and post-operative), yes/no | 9/17 ** | 8/13 ** | 0.86 | 0.22-3.37 | 1.00 |
| Aortic stenosis (pre- and post-operative), yes/no | 4/23 ** | 4/17 ** | 0.74 | 0.12-4.61 | 0.72 |
| Aortic stenosis (pre-operative), yes/no | 3/24 ** | 2/19 ** | 1.18 | 0.12-15.51 | 1.00 |
| Atrial fibrillation, yes/no | 5/31 | 9/26 ** | 0.47 | 0.11-1.80 | 0.25 |

OGIB, obscure gastrointestinal bleeding; OR, odds ratio; CI, confidence interval; IBD, inflammatory bowel disease; DOAC, direct oral anticoagulant; NSAIDs, non-steroidal anti-inflammatory drugs; PPI, proton pomp inhibitor; P-CAB, potassium-competitive acid blocker; WBC, white blood cells; Hb, hemoglobin; PT-INR, prothrombin time-international normalized ratio; BUN, blood urea nitrogen; Cr, creatinine; TP, total protein; Alb, albumin.

* Fisher’s exact test; ** Data excluding missing value; † Divided by median number.
