# Supplementary material for: Clinical features of obscure gastrointestinal bleeding undergoing capsule endoscopy: A retrospective cohort study
Source: PLoS One. 2022 Mar 24;17(3):e0265903. doi: 10.1371/journal.pone.0265903 (PMC8947120; doi:10.1371/journal.pone.0265903)
Supplement: S10 Table — (DOCX) [file pone.0265903.s012.docx]

**S10 Table. Comparison of clinical features of OGIB cases receiving different treatments, following propensity score matching**

| **Factors** | **Cases of OGIB** | | **Univariate** | | |
| --- | --- | --- | --- | --- | --- |
|  | Conservative treatment  (n = 30) | Endoscopic treatment, IVR, surgery (n = 30) | OR | 95% CI | *P* * |
| Presence of erosion or ulcer, yes/no | 10/20 | 11/19 | 1.16 | 0.35-3.83 | 1.00 |
| Presence of vascular lesions, yes/no | 4/26 | 14/16 | 5.52 | 1.41-27.16 | 0.010 |
| Current or former smoker, yes/no | 10/16 ** | 11/15 ** | 1.17 | 0.34-4.10 | 1.00 |
| Current warfarin user, yes/no | 2/28 | 4/26 | 2.13 | 0.28-25.39 | 0.67 |
| Current DOAC user, yes/no | 3/27 | 1/29 | 0.32 | 0.0057-4.21 | 0.61 |
| Current Aspirin user, yes/no | 7/23 | 3/27 | 0.37 | 0.056-1.86 | 0.30 |
| Current Thienopyridines user, yes/no | 3/27 | 0/30 | 0.00 | 0.00-2.37 | 0.24 |
| Current NSAIDs user, yes/no | 2/28 | 0/30 | 0.00 | 0.00-5.30 | 0.49 |
| Current probiotics user, yes/no | 5/23 ** | 1/29 | 0.16 | 0.0032-1.60 | 0.097 |
| Current PPI or P-CAB user, yes/no | 15/15 | 15/15 | 1.00 | 0.32-3.095 | 1.00 |
| WBC ≥ 5,080/µL, yes/no ^†^ | 13/16 ** | 12/18 | 0.82 | 0.26-2.61 | 0.63 |
| Hb ≥ 9.050 g/dL, yes/no ^†^ | 15/15 | 7/23 | 0.31 | 0.085-1.041 | 0.060 |
| Platelets ≥ 216.50/µL x10E3, yes/no ^†^ | 11/19 | 9/20 ** | 0.78 | 0.23-2.61 | 0.79 |
| PT-INR ≥ 1.075, yes/no ^†^ | 15/14 ** | 16/14 | 1.065 | 0.34-3.34 | 1.00 |
| BUN ≥ 14.80 mg/dL, yes/no ^†^ | 19/10 ** | 22/8 | 1.44 | 0.41-5.15 | 0.58 |
| Cr ≥ 0.80 mg/dL, yes/no ^†^ | 18/11 ** | 19/10 ** | 1.16 | 0.35-3.89 | 1.00 |
| BUN/Cr ≥ 16.83, yes/no ^†^ | 13/16 ** | 16/14 | 1.40 | 0.45-4.43 | 0.61 |
| TP ≥ 6.20 g/dL, yes/no ^†^ | 13/15 ** | 12/15 ** | 0.92 | 0.28-3.035 | 1.00 |
| Alb ≥ 3.30 g/dL, yes/no ^†^ | 12/16 ** | 15/15 | 1.33 | 0.42-4.26 | 0.61 |
| Hypertension, yes/no | 18/12 | 16/14 | 0.77 | 0.24-2.39 | 0.80 |
| Dyslipidemia, yes/no | 11/19 | 7/23 | 0.53 | 0.14-1.85 | 0.40 |
| Cerebral hemorrhage (current or past), yes/no | 2/28 | 1/29 | 0.49 | 0.0079-9.88 | 1.00 |
| Cerebral infarction (current or past), yes/no | 8/22 | 7/23 | 0.84 | 0.22-3.16 | 1.00 |
| Ischemic heart disease, yes/no | 5/25 | 5/25 | 1.00 | 0.20-4.94 | 1.00 |
| Valvulitis (pre- and post-operative), yes/no | 7/10 ** | 4/17 ** | 0.35 | 0.058-1.77 | 0.17 |
| Aortic stenosis (pre- and post-operative), yes/no | 4/14 ** | 1/20 ** | 0.18 | 0.0034-2.10 | 0.16 |
| Aortic stenosis (pre-operative), yes/no | 3/15 ** | 1/20 ** | 0.26 | 0.0045-3.59 | 0.32 |
| Atrial fibrillation, yes/no | 3/26 ** | 5/25 | 1.72 | 0.30-12.24 | 0.71 |

OGIB, obscure gastrointestinal bleeding; IVR, interventional radiology; OR, odds ratio; CI, confidence interval; IBD, inflammatory bowel disease; DOAC, direct oral anticoagulant; NSAIDs, non-steroidal anti-inflammatory drugs; PPI, proton pomp inhibitor; P-CAB, potassium-competitive acid blocker; WBC, white blood cells; Hb, hemoglobin; PT-INR, prothrombin time-international normalized ratio; BUN, blood urea nitrogen; Cr, creatinine; TP, total protein; Alb, albumin.

* Fisher’s exact test; ** Data excluding missing value; † Divided by median number.
